# Deoxynivalenol Exposure Leads to Abnormal Renal Tubular Autophagy Flow

**DOI:** 10.1002/advs.202505486

**Published:** 2025-09-26

**Authors:** Hao Chen, Xintong Zhou, Jun Ma

**Affiliations:** ^1^ College of Veterinary Medicine Northeast Agricultural University Harbin 150030 P. R. China; ^2^ Heilongjiang Provincial Key Laboratory of Pathogenic Mechanism for Animal Disease and Comparative Medicine Harbin 150030 P. R. China

**Keywords:** autophagy flux, deoxynivalenol, kidney, lysosome, mitochondria

## Abstract

Deoxynivalenol (DON) is one of the common mycotoxins contaminating feed. Renal tubular epithelial cells may experience oxidative stress as a result of consuming DON over time. However, little is known about the biological mechanisms through which DON results in kidney damage. This study employed proteomics, atomic force microscope (AFM), transmission electron microscope (TEM), and fluorescent probes, among other techniques, to reveal that DON exposure caused disturbances in renal autophagy flux both in vivo and in vitro. Mechanistically, this study found that DON exposure led to impaired formation of autophagosomes in the kidney. In addition, superoxide production by damaged mitochondria reduces ferroptosis resistance in renal tubules, and intracellular Fe^2+^ accumulates toward lysosomes, leading to impaired lysosomal function. This results in further disruption of autophagy flux. In summary, these findings substantiate the pivotal role of autophagy in renal injury induced by DON exposure, preliminarily elucidate the underlying mechanisms by which DON exposure triggers renal damage, and offer therapeutic strategies for its prevention and treatment.

## Graphical abstract

DON exposure disrupts the initiation of renal autophagy by inhibiting the formation of autophagosomes, thereby impairing mitochondrial degradation. Damaged mitochondria increase oxidative stress, reduce ferroptosis resistance, and cause lysosomal Fe^2^⁺ accumulation, further disrupting autophagy. These findings reveal DON's nephrotoxic mechanism and suggest potential therapeutic targets.

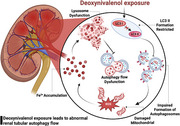

## 1 Introduction

Mycotoxin contamination of food and feed has always been a serious global problem, posing some risk to human health and the economy.^[^ ^1^ ^]^ Deoxynivalenol (DON) is one of the most common mycotoxins contaminating cereals and other crops.^[^ ^2^ ^]^ Exposure of humans and animals to DON results in a variety of acute and chronic toxic reactions, including enterotoxicity, nephrotoxicity, and hepatotoxicity.^[^ ^3^, ^4^, ^5^, ^6^ ^]^ DON is highly bioavailable, and it is rapidly absorbed in the gut, and the plasma circulation carries it to multiple organs.^[^ ^7^ ^]^ The kidney serves as the main metabolic organ for DON, but the toxicological mechanisms of DON on the kidney have not been adequately investigated. Oxidative stress is a major cause of DON‐induced renal injury and also serves as a major therapeutic target for mitigating DON‐induced renal injury.^[^ ^4^, ^8^ ^]^ In addition, oxidative stress injury is usually accompanied by changes in autophagy flow.^[^ ^9^ ^]^ Therefore, a better understanding of the role of autophagy in renal injury due to DON exposure will provide insight into the management of the “DON problem”.

The cargo is first surrounded by an isolation membrane to form autophagosomes, which subsequently fuse with lysosomes, and finally, the cargo is degraded. This process is called autophagy, which is essential for maintaining cellular homeostasis.^[^ ^10^ ^]^ In mammals, autophagosome formation, cargo recognition, and vesicle closure are dependent on autophagy related gene (ATG)8, and ATG8 has at least seven homologues (LC3, GABARAP, etc.).^[^ ^11^ ^]^ In addition, activation of ATG8 requires binding to phosphatidylethanolamine (PE), and this process requires coordination by ATG4B.^[^ ^12^ ^]^ ATG4B is both a conjugating and a deconjugating enzyme that ensures that ATG8 binds to the autophagosomal membrane.^[^ ^13^ ^]^ When autophagosomes bind to lysosomes, ATG4B is reactivated in order to delipidate and recycle ATG8.^[^ ^14^ ^]^ Although lipidation is important for ATG8 targeting the phagosome assembly site, delipidation of ATG8 is also required for efficient autophagy.^[^ ^15^ ^]^ It was shown that in yeast lacking ATG4‐mediated ATG8 delipidation, ATG8‐PE accumulates in various organelles and blocks autophagy.^[^ ^16^ ^]^ In vivo, the delipidating activity of ATG4B is closely related to the microenvironment in the cell, especially the level of reactive oxygen species (ROS).^[^ ^17^ ^]^ The interaction between Atg8 and PE also requires precise regulation by Atg4, ‐7, and ‐3, and the Atg12‐Atg5‐Atg16L1 complex. ATG8‐PE is closely associated with autophagosome development and maturation. Here, one of the objectives of this study is to investigate whether renal oxidative damage induced by DON exposure affects autophagosome formation.

Mitochondria are the energy‐producing hubs of the cell and consume the vast majority of the cell's oxygen. Cells remove senescent and damaged mitochondria by selective autophagy (mitophagy), which is prone to producing excessive ROS and triggering cellular damage.^[^ ^18^ ^]^ Mitochondrial ROS consist mainly of superoxide and H_2_O_2_, and superoxide is rapidly converted to H_2_O_2_, making the latter the main ROS released by mitochondria.^[^ ^19^ ^]^ Polyunsaturated fatty acids (PUFAs) in cell membranes are susceptible to damage by ROS, a process also known as lipid peroxidation. Excess lipid peroxidation increases cellular sensitivity to ferroptosis.^[^ ^20^ ^]^ Iron accumulation and lipid peroxidation play a major role in the initiation of ferroptosis; besides, the lysosome plays an important role in the cellular maintenance of iron homeostasis.^[^ ^21^ ^]^ Fe^3+^ bound to transferrin (Tf) enters the lysosome via the Tf receptor. In the lysosome, the ferric reductase STEAP3 converts Fe^3+^ to Fe^2+^, which is exported to the cytoplasm via the transporter DMT1. In addition, iron can enter the lysosome via ferritin binding to NCOA4.^[^ ^22^ ^]^ As the lysosome needs to accommodate high levels of Fe^2+^, this makes the lysosome more susceptible to oxidative damage.^[^ ^21^ ^]^ Renal oxidative damage by DON exposure has been demonstrated;^[^ ^23^ ^]^ however, its potential effect on autophagy flux remains unelucidated.

In this study, we demonstrated that DON exposure triggered renal tubular injury in mice. Further findings revealed that DON exposure impaired the formation of autophagosomes. In addition, we found that aberrant autophagy flow leads to the inability of damaged mitochondria to degrade and produce excess superoxide, which further triggers cellular ferroptosis and lysosomal dysfunction. Thus, our findings suggested that the autophagy process played a crucial role in protecting the kidney from DON exposure, which provides a potential target in the treatment of DON exposure‐induced injury.

## 2 Results

### 2.1 DON Exposure‐Induced Renal Tubular Injury in Mice

**Figure 1:**
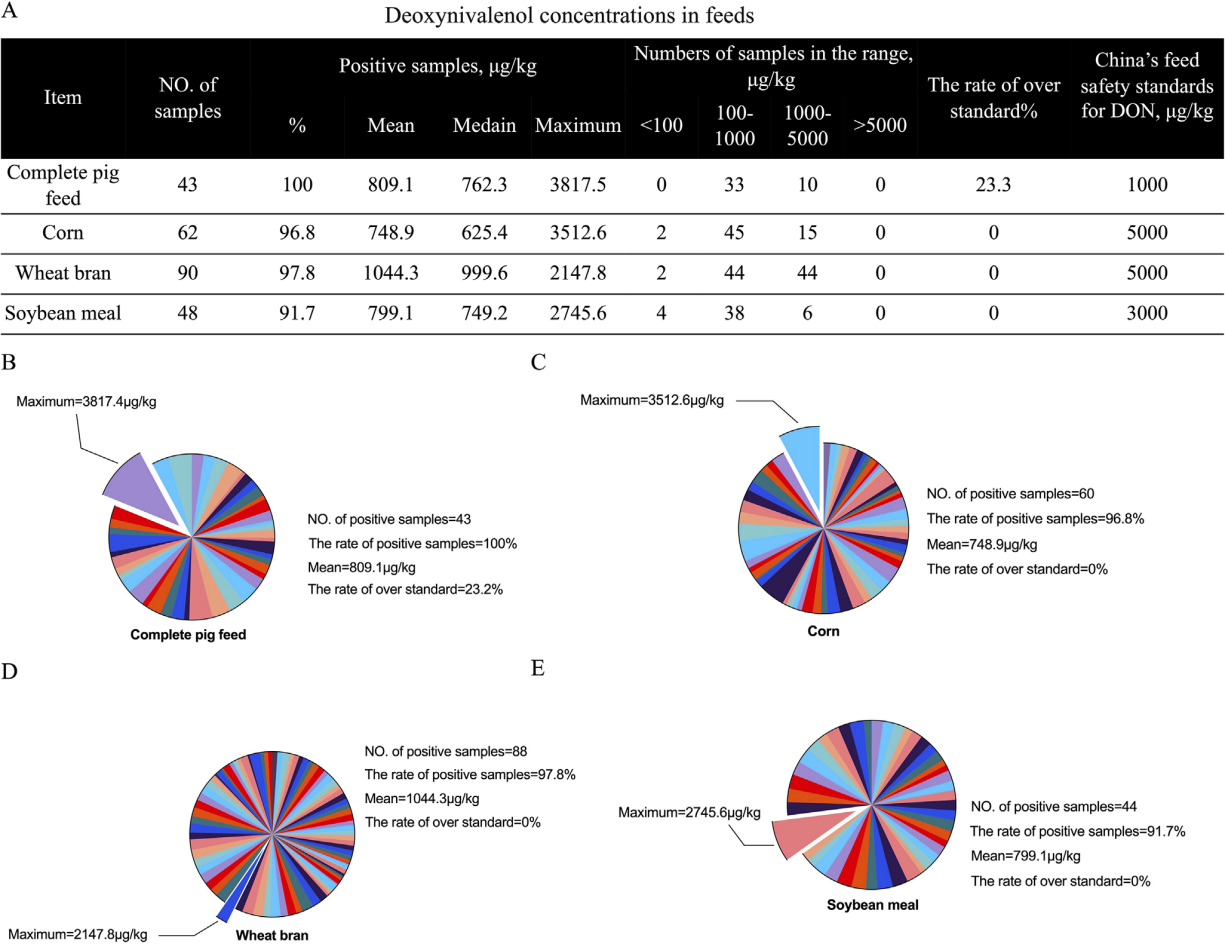
DON concentrations in feeds. A) DON concentrations in complete pig feed, corn, wheat bran, and soybean meal, where DON content greater than 100 µg kg^−1^ is recorded as a positive sample. B) DON in samples of complete pig feed, where the maximum concentration was 3817.4 µg kg^−1^. C) DON in samples of corn, where the maximum concentration was 3512.6 µg kg^−1^. D) DON in samples of wheat bran, where the maximum concentration was 2147.8 µg kg^−1^. E) DON in samples of soybean meal, where the maximum concentration was 2745.6 µg kg^−1^.

A total of 243 samples, including 43 complete feeds and 200 feedstuffs, were collected in this study. By analyzing the DON concentrations in the samples, DON were detected in 91.7%–97.8% of the feedstuffs and in 100% of the complete feeds (samples containing more than 100 µg kg^−1^ of DON were considered positive) (**Figure** **1**A). Ten samples were detected in complete feeds with DON concentrations exceeding the Chinese feed safety standard (DON concentrations <1000 µg kg^−1^). The maximum concentration of DON in complete feeds was 3817.5 µg kg^−1^, with an exceedance rate of 23.3% (Figure 1B). The highest mean DON concentrations of 1044.3 µg kg^−1^ were found in wheat bran (Figure 1D), followed by soybean meal (799.1 µg kg^−1^) (Figure 1E) and corn (748.9 µg kg^−1^) (Figure 1C).

**Figure 2:**
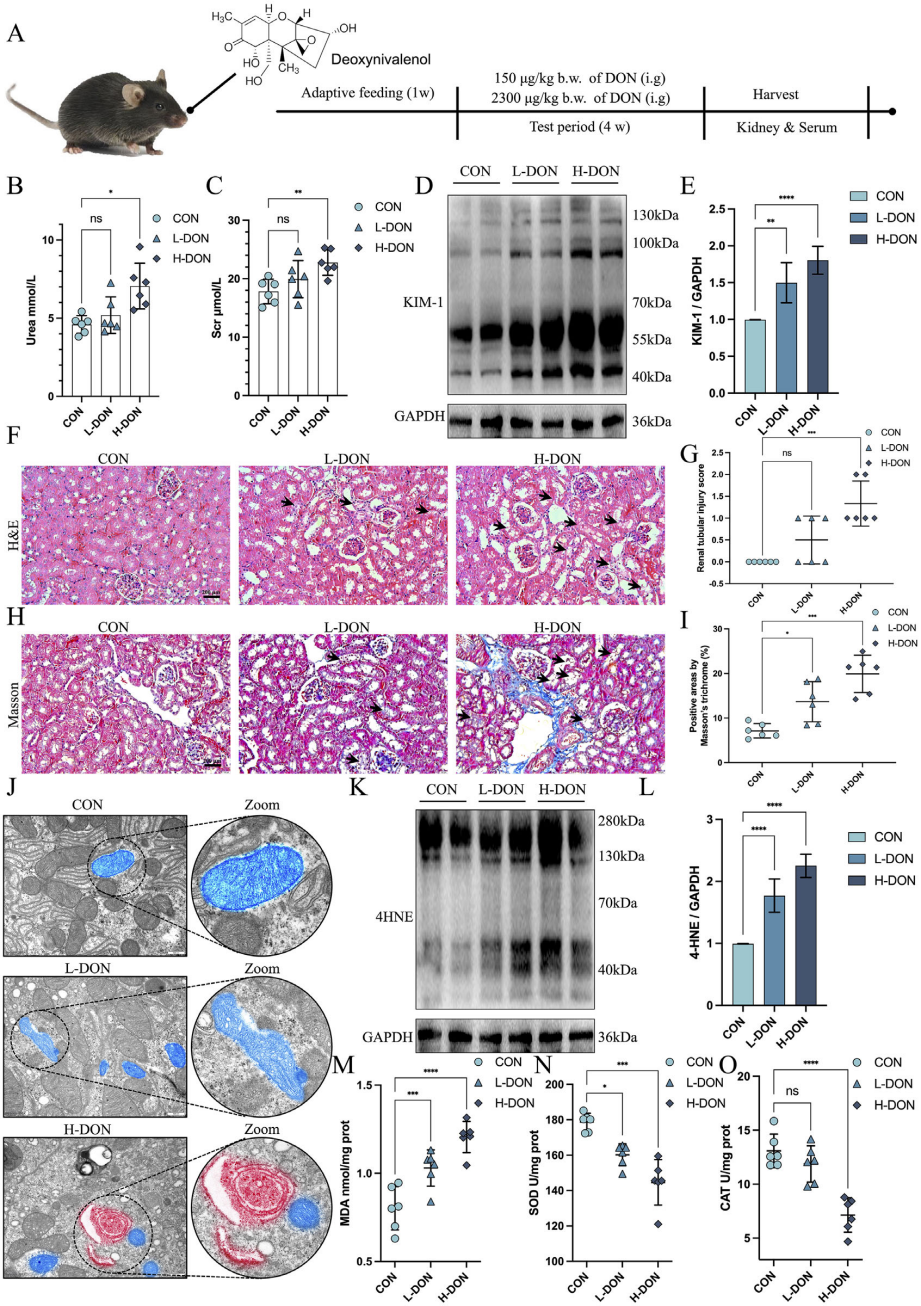
DON exposure induced renal tubular damage and ferroptosis in mice. A) Experimental period and drug dose. B, C) Serum Urea and Scr levels. D, E) Changes in KIM‐1 protein levels in the kidney detected by western blotting. F, G) Renal H&E staining pictures and renal tubular injury score (Scale bar = 200 µm). H, I) Renal Masson staining and positive areas by Masson staining (Scale bar = 200 µm). J) The representative TEM images of the kidney (Scale bar = 500 nm). The blue area was the mitochondria, and the red area was the isolation membrane. K, L) Changes in 4HNE protein levels in the kidney were detected by western blotting. M–O) MDA, SOD, and CAT contents of the kidney. The values were expressed as the mean ± S.D. (^****^ *P* < 0.0001 compared with the control group).

Considering the exceedance rate of complete feeds, the study was conducted for a 4‐week period in conjunction with the DON concentration in complete feeds (**Figure** **2**A). After 4 weeks of oral administration of DON to C57BL/6 mice, we observed changes in several biochemical parameters and pathological. As the experiment progressed, DON inhibited body weight growth and increased organ coefficients in the livers and kidneys of mice (Figure S1A–C, Supporting Information). High‐dose DON exposure could lead to elevated serum levels of Urea and Scr in mice. However, low‐dose DON exposure did not make a significant difference (Figure 2B,C). Due to the powerful metabolic functions of the kidneys, Urea and Scr are unable to detect kidney damage at an early stage. We examined the protein expression of KIM‐1 (an early marker of kidney injury) in the kidneys after DON exposure. Both L‐DON and H‐DON could significantly increase renal KIM‐1 protein levels (Figure 2D,E). In DON‐exposed mice, HE staining revealed detached renal tubular epithelial cell fragments and vacuole‐like degeneration (Figure 2F,G). Due to the limitations of tubular injury scoring, Masson staining could better observe the occurrence of renal fibrosis (Figure 2H,I). We wanted to dig further into how DON exposure leads to renal damage. We observed by TEM that DON reduced the size of mitochondria and blurred the mitochondrial bilayer membrane structure. Aggregation of proaulophagosomes toward mitochondria was even found after H‐DON exposure (Figure 2J). These changes in mitochondrial structure are consistent with the process by which ferroptosis occurs. DON exposure resulted in elevated protein levels of 4HNE (ferroptosis marker) in the renal compartment (Figure 2K,L), as well as causing alterations in markers of oxidative stress and lipid peroxidation (MDA, SOD and CAT) (Figure 2M–O). In addition, DON exposure caused an increase in TUNEL‐positive cells in the kidneys (Figure S1D, Supporting Information). Based on these results, we hypothesized that DON exposure could lead to a trend toward ferroptosis in the mouse kidney.

### 2.2 DON Exposure Induced Ferroptosis in TCMK‐1

**Figure 3:**
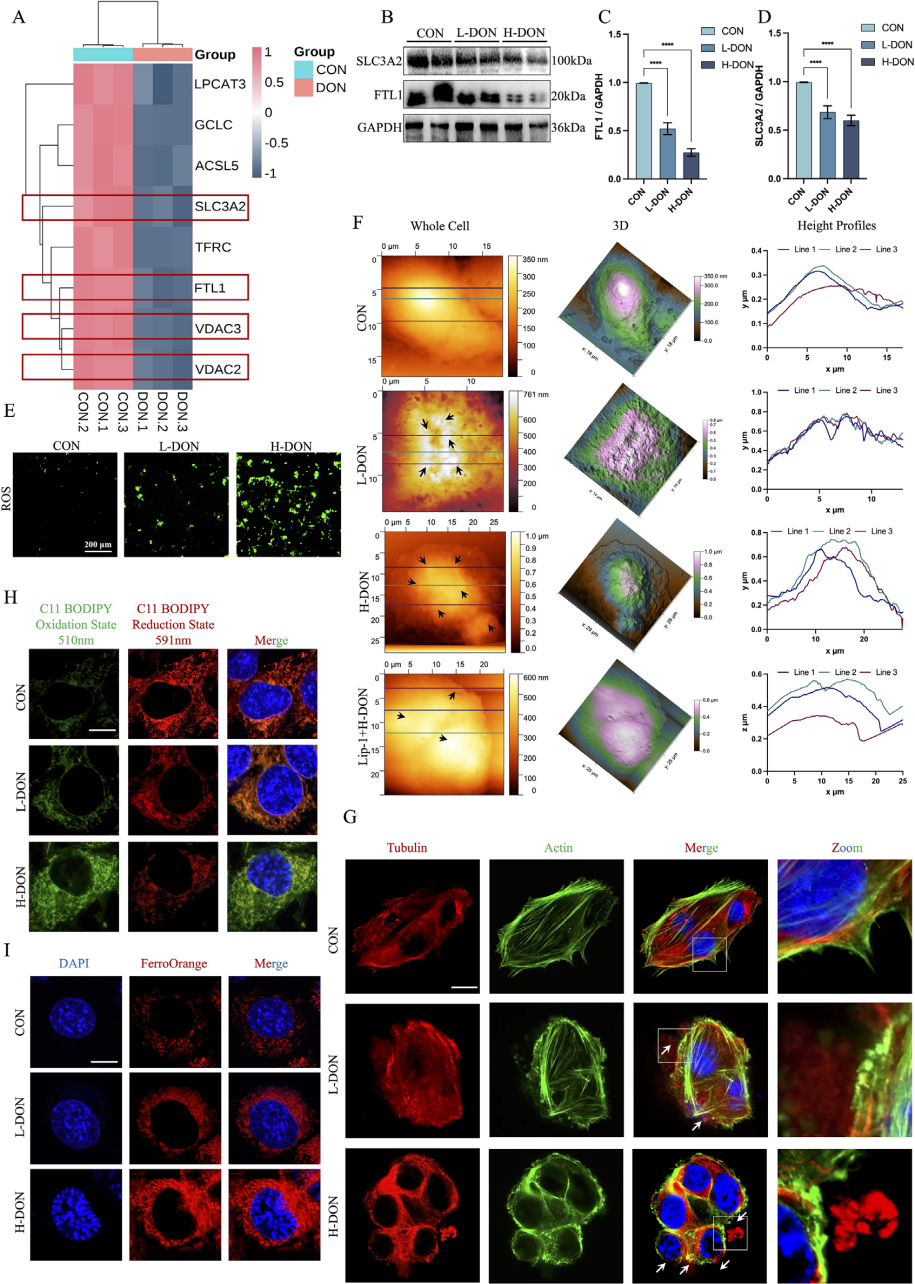
DON exposure induced ferroptosis in TCMK‐1. A) DON treatment of TCMK‐1 was followed by proteomic analysis. Heatmap of 8 proteins associated with ferroptosis in the H‐DON groups compared to the control groups (n = 3 technical replicates). B–D) TCMK‐1 was treated with 100 or 200 ng mL^−1^ DON for 48 h. SLC3A2 and FTL1 levels were detected by immunoblotting with antibodies. E) ROS levels in TCMK‐1. F) Three morphologically characterized images: left, 2D AFM image; middle, 3D AFM image; right, profile of the typical characteristic geometry of RCD. G) Stained TCMK‐1 with Actin and Tubulin probes were observed by confocal microscopy (Scale bar = 10 µm). Membrane blebs could be distinguished (white arrows). H) Stained TCMK‐1 with C11 BODIPY was observed by confocal microscopy (Scale bar = 10 µm). I) Stained TCMK‐1 with FerroOrange was observed by confocal microscopy (Scale bar = 10 µm). The values were expressed as the mean ± S.D. (^*^ *P* < 0.05, ^**^ *P* < 0.01, ^***^ *P* < 0.001, and ^****^ *P* < 0.0001 compared with the control group).

To investigate the protein level changes that occur in mouse renal tubules as a result of DON exposure. We performed proteomics of TCMK‐1 after DON treatment (Figure S2A,B, Supporting Information). We identified 1858 major protein IDs from TCMK‐1 and normalized protein intensities between samples and analyzed them by KEGG (Figure S2C, Supporting Information). KEGG analysis showed a significant increase in pathways related to programmed cell death after DON treatment, including autophagy, mitophagy, and ferroptosis (Figure S2D, Supporting Information). The enrichment results were particularly significant in ferroptosis, and DON treatment caused downregulation of SLC3A2, FTL1, VDAC3 and VDAC2 protein levels in TCMK‐1 (**Figure** **3**A). Meanwhile, we found that DON treatment led to downregulation of SLC3A2 and FTL1 protein levels in TCMK‐1 by immunoblotting (Figure 3B–D) This indicated that DON could cause impairment of the X_C‐_system and degradation of ferritin in TCMK‐1. In addition, DON caused elevated ROS levels in TCMK‐1 and reduced the antioxidant capacity of TCMK‐1 (Figures 3E; S3A, Supporting Information). In order to more visually observe the effect of DON exposure on TCMK‐1 morphological changes, we performed morphological scans of TCMK‐1 using AFM. One could notice that 0.4–0.8 µm protrusions were formed on the cell membrane of TCMK‐1 after DON treatment (Figure 3F). These irregular protrusions might be caused by lipid peroxide accumulation leading to cell membrane instability. Therefore, we examined whether DON exposure could cause TCMK‐1 lipid peroxidation using the Bodipy C11 probe. The results showed that DON changed the Bodipy C11 probe from a reduced state to an oxidized state in TCMK‐1 (Figures 3H; S3B, Supporting Information). Furthermore, Liproxstatin‐1 effectively alleviated DON‐induced cellular morphological alterations (Figure 3F), reduced lipid peroxidation levels (Figure S3C,D, Supporting Information), and enhanced the proliferation of TCMK‐1 (Figure S3E, Supporting Information). Since extensive lipid peroxidation is a key downstream feature of iron death, we hypothesized that DON exposure could lead to TCMK‐1 ferroptosis. When cells undergo a tendency toward ferroptosis, the cytoskeletal organization is also altered to some extent. To verify that DON exposure induces TCMK‐1 to undergo ferroptosis, we analyzed the two main components of the cytoskeleton (actin and tubulin) using immunofluorescence. DON caused rounding of TCMK‐1 cells. And tubulin protruded from the surface of the cell membrane with no surrounding wrapping actin (Figure 3G). This might be due to the excessive accumulation of Fe^2+^ in TCMK‐1 under DON exposure (Figures 3I; S3F, Supporting Information), which led to the weakening of the antioxidant capacity of the membrane structure and triggered the rupture of the cell membrane.

### 2.3 DON Exposure Contributed to Blocked Autophagy Flow and Mitochondrial Dysfunction in TCMK‐1

**Figure 4:**
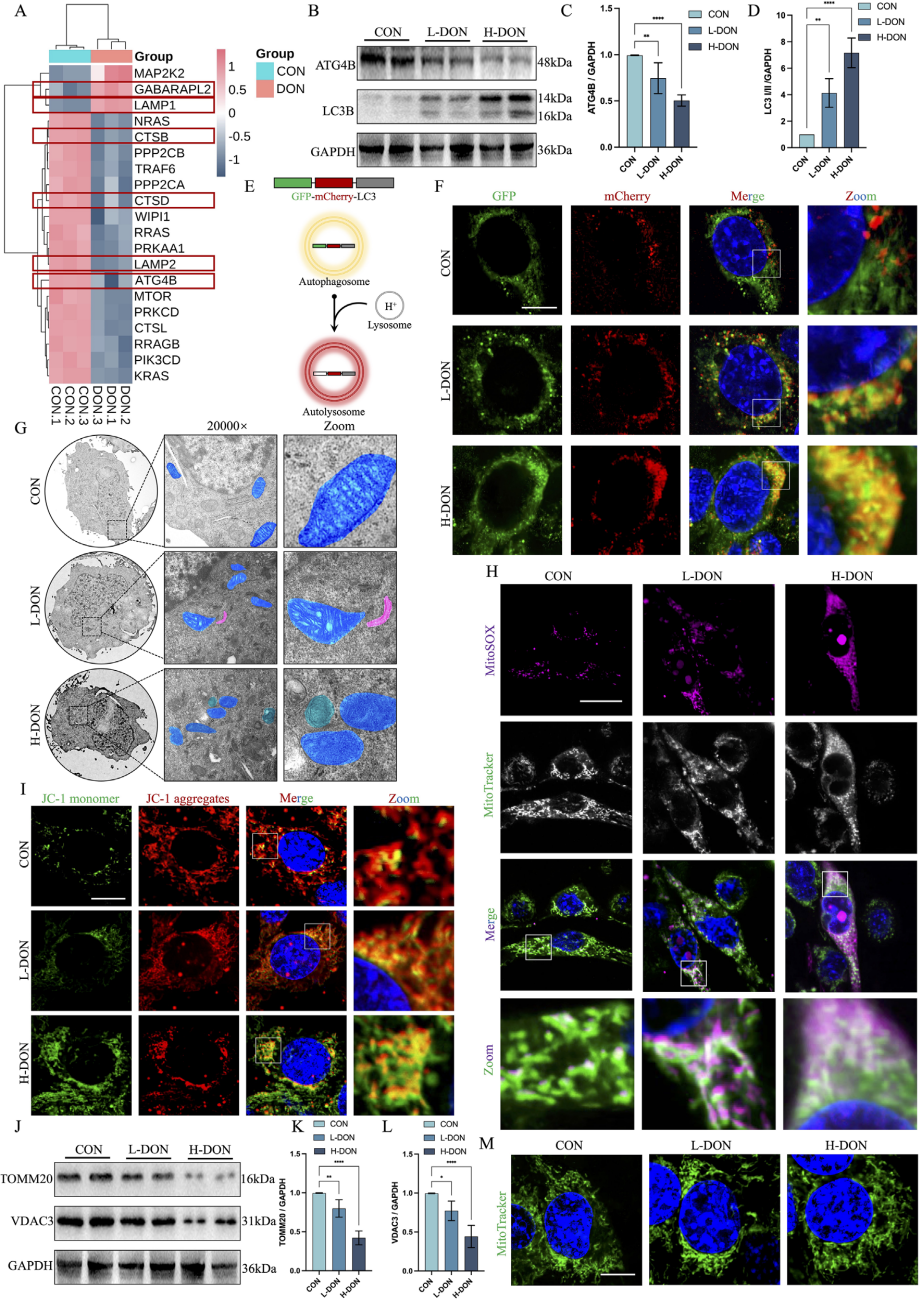
DON exposure caused impaired TCMK‐1 autophagy flow and induced mitochondrial dysfunction. A) DON treatment of TCMK‐1 was followed by proteomic analysis. Heatmap of 20 proteins associated with autophagy in the H‐DON groups compared to the control groups (n = 3 technical replicates). B–D) ATG4B and LC3 levels were detected by immunoblotting with antibodies. E, F) Representative images of TCMK‐1‐transfected Ad‐mCherry‐GFP‐LC3B in the presence of DON treatment. G) The representative TEM images of TCMK‐1 (Scale bar = 500 nm). The blue area was the mitochondria, the red area was the isolation membrane, and the green area was the autophagosome. H) Stained TCMK‐1 with MitoSOX and MitoTracker was observed by confocal microscopy (Scale bar = 10 µm). I) Stained TCMK‐1 with JC‐1 probe was observed by confocal microscopy (Scale bar = 10 µm). J–L) TOMM20 and VDAC3 levels were detected by immunoblotting with antibodies. M) Stained TCMK‐1 with MitoTracker was observed by confocal microscopy (Scale bar = 10 µm). The values were expressed as the mean ± S.D. (^*^ *P* < 0.05, ^**^ *P* < 0.01, and ^****^ *P* < 0.0001 compared with the control group).

The results of KEGG analysis similarly demonstrated that DON exposure could lead to abnormal TCMK‐1 autophagy. By analyzing the results, we found an interesting phenomenon: DON exposure resulted in the up‐regulation of GABAPAPL2 and down‐regulation of ATG4B protein levels in TCMK‐1 (**Figure** **4**A). By immunoblotting, we got similar results. DON exposure upregulated LC3 I/II protein levels and downregulated ATG4B protein levels in TCMK‐1 (Figure 4B–D). However, the accumulation of LC3 I was more significant, indicating that the conversion of LC3 I to LC3 II was partially impaired. Therefore, we attempted to detect whether the autophagy flux in TCMK‐1 was abnormal. Given the property that GFP is quenched under acidic conditions (Figure 4E), we used GFP‐mCherry‐LC3B to detect autophagy flow in TCMK‐1 under DON exposure. DON exposure reduced the proportion of autolysosome numbers (red puncta) in TCMK‐1 compared to control cells (Figures 4F; S4A,B, Supporting Information). This suggested that DON exposure has the potential to affect autophagy flow and autophagy initiation in TCMK‐1. It should be noted that STK683963 (ATG4B agonist) failed to completely restore the autophagy flux impairment caused by DON exposure (Figure S4C–E, Supporting Information). Chloroquine (CQ) did not further increase the number of autophagosomes in the H‐DON group. These results strongly suggested that DON exposure caused abnormalities downstream of the autophagy flux in TCMK‐1 (Figure S4F–H, Supporting Information). In addition, TEM images showed that DON exposure could lead to structural disorganization of mitochondrial ridges, volume reduction, and gradual loss of bilayer membrane structure in TCMK‐1. And the L‐DON groups found that the isolation membrane appeared near the mitochondria. Although mitophagy occurred in the H‐DON groups, the number of mitochondria with non‐normal structures was significantly greater than those engulfed by autophagosomes (Figure 4G).

Therefore, we wondered whether the elevated ROS levels in TCMK‐1 by DON exposure were related to the inability of cells to clear damaged mitochondria. In this study, changes in TCMK‐1 mitochondrial membrane potential and mitochondrial superoxide after DON exposure were detected by the JC‐1 and MitoSOX probes. Unsurprisingly, DON exposure led to an increase in mitochondrial superoxide levels (Figures 4H; S5A,B, Supporting Information), a decrease in mitochondrial membrane potential (Figures 4I; S5C, Supporting Information), and an inhibition of ATP synthesis (Figure S5D,E, Supporting Information) in TCMK‐1, implying that DON exposure triggered TCMK‐1 mitochondrial damage. Analysis of KEGG results indicated downregulation of TOMM20 and VDAC3 protein levels located in the outer mitochondrial membrane in DON‐exposed TCMK‐1 cells (Figure S6A, Supporting Information; Figure 3A). Immunoblotting verified changes in TOMM20 and VDAC3 protein levels in TCMK‐1 after DON exposure (Figure 4J–L). Localization of mitochondria in TCMK‐1 using the MitoTracker probe similarly demonstrated that DON exposure impaired the TCMK‐1 mitochondrial network (Figures 4M; S5F,G, Supporting Information).

### 2.4 MitoTempo Could not Completely Mitigate Ferroptosis Caused by DON Exposure in TCMK‐1

**Figure 5:**
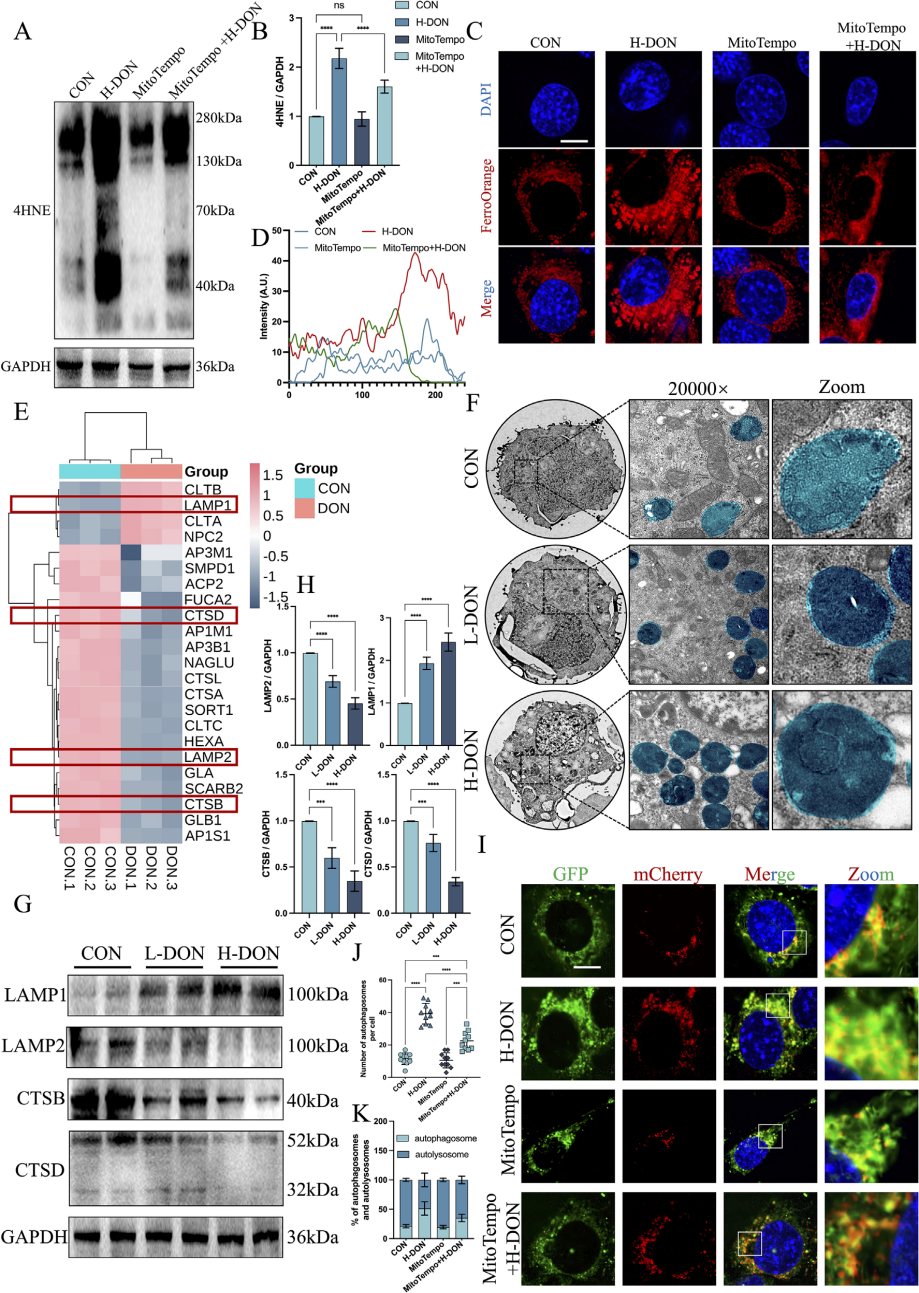
DON exposure induced TCMK‐1 lysosomal dysfunction. A, B) TCMK‐1 were treated with 200 ng mL^−1^ DON for 48 h, followed by replacing the medium containing MitoTempo (10 µM) for 24 h. 4HNE levels were detected by immunoblotting with antibodies. C, D) Stained TCMK‐1 with FerroOrange was observed by confocal microscopy (Scale bar = 10 µm). E) Heatmap of 23 proteins associated with the lysosome in the H‐DON groups compared to the control groups (n = 3 technical replicates). F) The representative TEM images of TCMK‐1 (Scale bar = 500 nm). The blue area was the lysosome. G, H) LAMP1, LAMP2, CTSB, and CTSD levels were detected by immunoblotting with antibodies. I–K) Representative images of TCMK‐1 transfected Ad‐mCherry‐GFP‐LC3B in the presence of DON or MitoTempo treatment. The number of green‐red positive puncta in each cell was determined, and the ratio of autophagosomes to autolysosomes was calculated. The values were expressed as the mean ± S.D. (^***^ *P* < 0.001 and ^****^ *P* < 0.0001 compared with the control group).

Based on the above results, we tried to antagonize the toxicity of DON on TCMK‐1 using MitoTempo (a mitochondrial antioxidant). Interestingly, MitoTempo was unable to completely alleviate DON exposure‐induced TCMK‐1 ferroptosis. These results included the changes in 4HNE protein levels (**Figure** **5**A,B), the TUNEL analysis (Figure S6B, Supporting Information), and the extent of Fe^2+^ accumulation (Figure 5C,D) in TCMK‐1. This would mean that there were other factors involved in TCMK‐1 damage induced by DON exposure. In KEGG analysis, we noted that DON exposure triggered lysosomal abnormalities in TCMK‐1, including upregulation of LAMP1 protein levels and downregulation of LAMP2, CTSB, and CTSD protein levels (Figure 5E). In the TEM results, we observed lysosome accumulation in TCMK‐1 after DON exposure (Figure 5F). Similarly, immunoblotting results showed that DON exposure resulted in increased expression of LAMP1 protein levels and downregulation of lysosomal function‐related proteins in TCMK‐1 (Figure 5G,H). DON exposure increased LAMP2 mRNA levels but suppressed LAMP2 protein levels (Figure S6C, Supporting Information). These results suggested that DON not only affects the autophagy initiation phase of TCMK‐1 but also inhibits lysosome function, further inhibiting the autophagy flow of TCMK‐1. Even after MitoTempo treatment, some autophagosomes were still present in TCMK‐1, further proving that DON exposure could lead to lysosomal dysfunction in TCMK‐1 (Figure 5I–K).

### 2.5 DON Exposure Caused Fe^2+^ Accumulation‐Mediated Lysosomal Dysfunction in TCMK‐1

**Figure 6:**
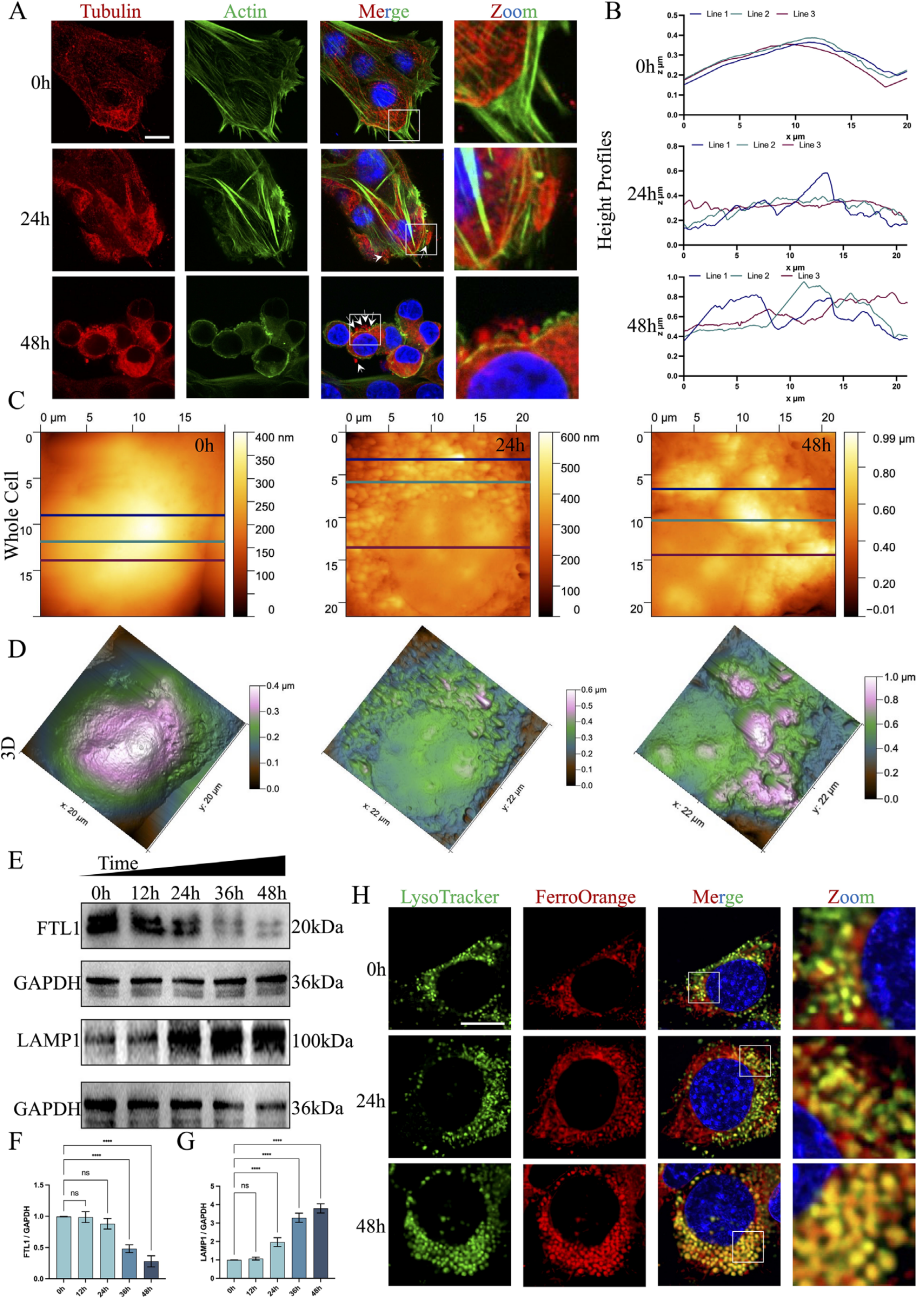
DON exposure caused Fe^2+^ to aggregate toward lysosomes in TCMK‐1. A) TCMK‐1 was treated with 200 ng mL^−1^ DON for 24 or 48 h. Stained TCMK‐1 with Actin and Tubulin probes were observed by confocal microscopy (Scale bar = 10 µm). Membrane blebs could be distinguished (white arrows). B) Profile of the typical characteristic geometry of RCD. C) 2D AFM image. D) 3D AFM image. E–G) TCMK‐1 were treated with 200 ng mL^−1^ DON for 12, 24, 36, or 48 h. FTL1 and LAMP1 levels were detected by immunoblotting with antibodies. H) Stained TCMK‐1 with LysoTracker and FerroOrange was observed by confocal microscopy (Scale bar = 10 µm). The values were expressed as the mean ± S.D. (^****^ *P* < 0.0001 compared with the control group).

We attempted to identify the cause of TCMK‐1 lysosomal dysfunction due to DON exposure. Because cellular ferroptosis could induce lysosomal dysfunction,^[^ ^24^ ^]^ we speculated whether DON‐induced lysosomal dysfunction was ferroptosis‐mediated. First, H‐DON treated TCMK‐1 for 24 and 48 h. We examined cytoskeletal changes (**Figure** **6**A) and cell surface morphology of TCMK‐1 (Figure 6B–D), respectively. The results showed that DON exposure resulted in gradual “ferroptosis‐like” morphological changes in TCMK‐1. Subsequently, H‐DON treated TCMK‐1 for 12, 24, 36, and 48 h. Immunoblotting results showed a gradual decrease in FTL protein levels over time and a gradual increase in LAMP1 protein levels (Figure 6E–G). This suggested that Fe^2+^ accumulation was concomitant with impaired lysosome accumulation in TCMK‐1. In addition, protein levels of lysosomal function‐related proteins (LAMP2, CTSB, CTSD) decreased with prolonged DON exposure (Figure S7A–D, Supporting Information). The iron chelator Deferoxamine B (DFO) could effectively alleviate DON exposure‐induced CTSB and CTSB protein level decreases in TCMK‐1 (Figure S7E–G, Supporting Information). In order to more intuitively observe the relationship between changes in Fe^2+^ content and lysosomes in TCMK‐1, we used both FerroOrange and LysoTracker probes. Fe^2+^ in TCMK‐1 gradually bonded to lysosomes with increasing DON exposure time (Figure 6H), and the number of lysosomes gradually multiplied (Figure S7H–J, Supporting Information). Also, DFO could reduce the increase in the number of lysosomes caused by DON exposure (Figure S7K–N, Supporting Information). These results might imply that DON exposure triggers TCMK‐1 to undergo ferroptosis‐mediated lysosomal dysfunction.

### 2.6 DON Exposure Induced Renal Lysosomal Dysfunction in Mice

**Figure 7:**
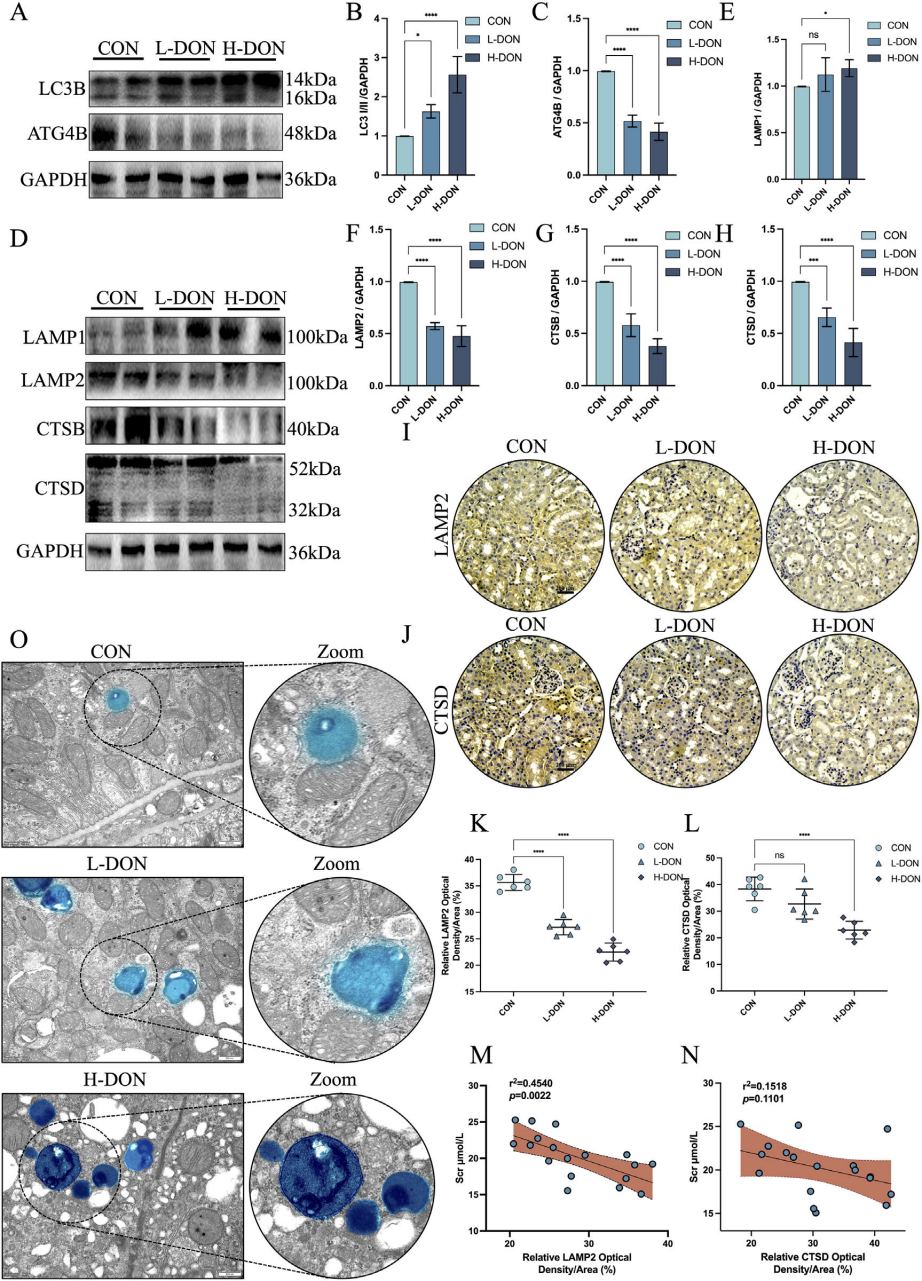
DON exposure induced renal lysosomal dysfunction in mice. A–C) Changes in LC3 and ATG4B protein levels in the kidney were detected by western blotting. D–H) Changes in LAMP1, LAMP2, CTSB, and CTSD protein levels in the kidney were detected by western blotting. D, K) IHC staining for LAMP2 in the kidney (Scale bar = 200 µm). J, L) IHC staining for CTSD in the kidney (Scale bar = 200 µm). O) The representative TEM images of the kidney (Scale bar = 500 nm). The blue area was the lysosome. M) Correlation between the density of LAMP2 immunostaining and Scr in the mouse kidney. N) Correlation between density of CTSD immunostaining and Scr in the mouse kidney. The values were expressed as the mean ± S.D. (^*^ *P* < 0.05, ^***^ *P* < 0.001, and ^****^ *P* < 0.0001 compared with the control group).

In order to verify the potential mechanism of renal tubular injury in mice caused by DON exposure, we conducted a series of in vivo studies in mice. By immunoblotting, we detected a significant increase in LC3I/II protein levels (**Figure** **7**A,B) and a dramatic decrease in ATG4B protein levels (Figure 7A,C) in the kidneys of DON‐exposed mice. This would imply that DON exposure affects the initiation of renal autophagy in mice. Next, we examined the expression levels of lysosome‐associated proteins in the kidneys of mice after DON exposure. The results showed that DON exposure significantly upregulated LAMP1 protein expression levels in the kidney (Figure 7D,E). Furthermore, DON exposure downregulates protein levels of lysosomal function‐related proteins (LAMP2, CTSB, and CTSD) in mice kidneys (Figure 7D,F–H). Normally, deficiency of CTSD and LAMP2 directly affects lysosomal function and impairs the autophagosome‐lysosome machinery. We stained CTSD (Figure 7J,L) and LAMP2 (Figure 7I,K) in the kidneys for both and explored the relationship between CTSD and LAMP2 expression and renal tubular injury in mice. The findings of the analysis showed a moderate correlation between the density of CTSD (r^2^ = 0.4540, *p* = 0.0022) and LAMP2 (r^2^ = 0.1518, *p* = 0.1101) and serum creatinine (Figure 7M,N). Meanwhile, we observed the number of lysosomes in the kidneys of mice after DON exposure using TEM. More lysosomes were present in the kidneys of DON‐exposed mice compared to the controls (Figure 7O). The above results demonstrated once again that DON exposure caused impaired renal lysosomal function in mice.

## 3 Discussion

Mycotoxins are toxic metabolites produced by fungi such as *Aspergillus*, *Penicillium*, *Fusarium*, and *Alternaria*. The “from farm to fork” principle highlights how easily mycotoxins can enter the human food chain. Currently, researchers have found that aflatoxin B1, zearalenone, DON, and other mycotoxins can cause liver toxicity, kidney toxicity, reproductive toxicity, and other adverse effects.^[^ ^25^, ^26^ ^]^ Notably, the contamination rate of DON is significantly higher than that of other mycotoxins. The “DON problem” is becoming a “DON challenge” for farmers, consumers, and producers.^[^ ^27^ ^]^ The “DON problem” is likely to get worse as a result of climate change and increasing demand for food and feed.^[^ ^28^ ^]^ In particular, DON contamination in feed has a negative effect on growth performance as well as organ function in pigs.^[^ ^29^ ^]^ An investigative study showed that the detection rate of DON in feed samples (feed ingredients and complete feeds) ranged from 96.4% to 100% in different regions of China.^[^ ^30^ ^]^ We were limited in the number of samples tested as well as the number of years, but the detection rate of DON did not differ significantly from their results. It is worth mentioning that although the DON content in complete feeds exceeds the Chinese safety standard at a higher rate, we should be aware that feed ingredients are also contaminated with DON. Therefore, optimizing the management of the supply chain system is also the “DON problem” to be faced.^[^ ^27^ ^]^ Even if DON levels are relatively low, feeding DON‐contaminated diets for extended periods of time can affect the accumulation of DON in the muscles and organs of pigs.^[^ ^31^ ^]^ In addition, consumption of this pork is one of the main routes of human exposure to DON. DON has a high bioavailability of ≈45%–100% in rats, humans, and pigs.^[^ ^7^ ^]^ Following oral administration of DON, DON is first concentrated in the plasma and then rapidly distributed to multiple organs. The highest levels of DON in each organ were found in plasma, kidney, and small intestine.^[^ ^32^, ^33^ ^]^ The kidney is the main metabolizing organ for DON, so renal injury from DON exposure should receive more attention. The small intestinal epithelium is highly sensitive to DON, and DON exposure causes an imbalance in the intestinal flora, thereby causing sustained negative effects on the organism.^[^ ^5^ ^]^ Many studies of DON exposure to kidney and small intestine damage point to excessive accumulation of ROS, which triggers a series of programmed necrosis (e.g., iron death, apoptosis).^[^ ^23^, ^34^ ^]^ A closely related connection exists between the kidneys and the intestines, which is referred to as the gut‐kidney axis. Disturbances in the intestinal flora increase the production of urinary toxins, and kidney damage similarly affects the abundance of intestinal flora.^[^ ^35^ ^]^ We conjecture that kidney damage from DON exposure interacts with intestinal flora disruption in a vicious cycle. Although most of the previous damage caused by DON exposure has been focused on the intestinal, in this study, we would like to illustrate the toxicity of DON from a renal damage perspective.

Among the four ATG4 homologues in mammals, ATG4B plays a dominant role in the regulation of autophagy because ATG4B cleaves all homologues of ATG8.^[^ ^36^ ^]^ This means that ATG4B controls the activity of all ATG8 homologues. Accumulation of ROS in the cell can directly lead to elevated H_2_O_2_, the oxidative signal that inactivates ATG4B.^[^ ^17^ ^]^ Thus, inactivated ATG4B inhibited the delipidation and recycling of ATG8.^[^ ^37^ ^]^ As reflected in our study, DON elevated ROS in TCMK‐1, and the intracellular hyperoxidative state reduced ATG4B activity. However, ATG4B has the dual function of promoting LC3 lipidation and delipidation.^[^ ^13^ ^]^ For instance, the small molecule compound NSC185058 was shown to reduce the accumulation of LC3‐PE in SAOS‐2.^[^ ^38^ ^]^ These findings are controversial, suggesting that the function of ATG4B depends on the specific situation. Following DON treatment, the accumulation of LC3 I in TCMK‐1 indicated that the ability of Atg4B to support Atg8 lipidation was not affected. The finding that LC3 II accumulation was less pronounced than LC3 I accumulation indicates defective autophagosome formation. We hypothesized that this could not be solely attributed to the inhibition of ATG4B activity. This may be closely related to the impeded transition from LC3 I to LC3 II. Research indicated that DON exposure also down‐regulated ATG5 and ATG7 protein expression.^[^ ^6^ ^]^ Notably, STK683963 could not fully restore the TCMK‐1 autophagy flux impairment caused by DON exposure. This finding further indicates that DON‐induced impairment of autophagosome formation in TCMK‐1 exhibits multidimensionality. The mammals have six ATG8 homologues, and because of the differences in conformation, they are grouped into the LC‐family and the GABARAP‐family.^[^ ^11^ ^]^ Since ATG4B treats LC3I/II over 1500‐fold more efficiently than other ATG4 homologues, and ATG4A is more efficient at treating GABARAP‐L2.^[^ ^39^ ^]^ Therefore, we verified the protein expression of LC3I/II but not GABARAP‐L2 in TCMK‐1. This also points out the limitations of our findings.

Blocking autophagy has a minimal effect on cell death but increases the accumulation of cellular debris.^[^ ^40^ ^]^ This suggests that blocking autophagy in cells inhibits the degradation of cellular debris and accelerates the process of cell death. For instance, inhibition of autophagy in the nervous system disrupts cellular homeostasis and results in dysfunctional mitochondria that are unable to degrade.^[^ ^41^ ^]^ In our current experimental setting, DON exposure disrupted the autophagy flow of TCMK‐1, which in turn resulted in dysfunctional mitochondria that could not be degraded. We suggested that the accumulation of mitochondrial superoxide directly disrupts the oxidative homeostasis of the cell. This caused oxidative damage to PUFAs, which in turn led to reduced sensitivity of TCMK‐1 to ferroptosis. The antiporter X_C‐_, encoded by the SLC7A11 gene, is a key regulator of cellular redox mechanisms that protect cells against lipid peroxidation.^[^ ^42^ ^]^ SLC3A2 is a chaperone protein of SLC7A11 that serves to support SLC7A11 function, and it is equally important for X_C‐_ system function.^[^ ^43^, ^44^ ^]^ Impaired function of the X_C‐_system leads to diminished cellular resistance to lipid peroxidation.^[^ ^42^ ^]^ In this study, we reported that DON exposure down‐regulates protein levels of SLC3A2 in mice kidney. Meanwhile, DON degraded FTL proteins in mice's kidneys and TCMK‐1, and enhanced accumulation of Fe^2+^ and lipid peroxidation were observed. Although DON exposure caused impaired autophagy flux in TCMK‐1, we propose that FTL1 protein degradation still occurs via residual autophagy flux, leading to Fe^2+^ accumulation within TCMK‐1. This would imply that DON exposure led to ferroptosis in the kidneys of mice, which might be due to a combination of decreased renal resistance to lipid peroxidation and Fenton reaction‐mediated Fe^2+^ and peroxides. In addition, we also noted that DON increased the products.^[^ ^45^ ^]^ of lipid peroxidation of polyunsaturated fatty acids (4HNE) in mice kidney, which is one of the strongest pieces of evidence that DON caused ferroptosis in mice kidney.

Many harmful reactions in the organism require the participation of iron and occur in the lysosome.^[^ ^46^ ^]^ Together with H_2_O_2_ dispersed into the lysosome, it triggers the Fenton reaction, which leads to a homeostatic loss of the lysosome.^[^ ^47^ ^]^ This also explains why the lysosome is more sensitive to oxidative stress damage than other organelles. In the present study, we first found that DON exposure led to an increase in the expression of the lysosomal marker LAMP1 protein and subsequently observed a decrease in the expression of the CTSB and CTSD proteins associated with the degradation of cargo by the autolysosome. LAMP2 is a highly glycosylated protein that is critical for lysosomal function. Inhibition of N‐glycosylation causes a decrease in LAMP2 expression and leads to lysosomal accumulation and dysfunction.^[^ ^48^ ^]^ Although DON can induce changes in O‐GlcNAcylation in liver,^[^ ^49^ ^]^ whether it affects N‐glycosylation remains to be investigated. This indicated that DON exposure led to lysosomal dysfunction in the kidneys of mice and again explained the blockage of autophagy flow. Notably, DMT1 plays an important role in maintaining Fe^2+^ homeostasis in the lysosome, and inactivation of DMT1 can lead to ferroptosis‐dependent lysosomal dysfunction.^[^ ^24^ ^]^ Whether DON exposure regulates DMT1 activity is also something we need to investigate in the future. The lysosome plays a key role in cellular iron transport and homeostasis.^[^ ^50^ ^]^ TRPML1 is a non‐selective cation channel that's specifically found in lysosomes and helps them work by keeping the ion balance right in different types of cells. From a functional perspective, TRPML1 channels can regulate Fe^2+^ exocytosis and endocytosis in lysosomes.^[^ ^51^ ^]^ Damaged lysosomes may cause impaired TRPML1 channel function, leading to Fe^2+^ accumulation within lysosomes. DON exposure may exacerbate damage to already compromised lysosomes through this mechanism, but the specific pathway remains to be elucidated. Abnormalities in iron metabolism are also strongly associated with renal tubular injury.^[^ ^52^ ^]^ In a diabetic rat model, excess Fe^2+^ accumulated in the lysosome within proximal renal tubular epithelial cells, triggering renal tubular injury. In addition, iron accumulation in the lysosome was positively correlated with renal tubular injury.^[^ ^53^ ^]^ In this study, we explored the preliminary mechanisms of DON exposure‐induced kidney injury in mice, but the relationship between the various negative biological processes triggered by DON remains unclear. Notably, the effects of DON exposure on gut flora have received similar attention. Therefore, future research will focus on the intrinsic mechanisms of damage to the gut‐kidney axis by DON exposure.

## 4 Conclusion

**Figure 8:**
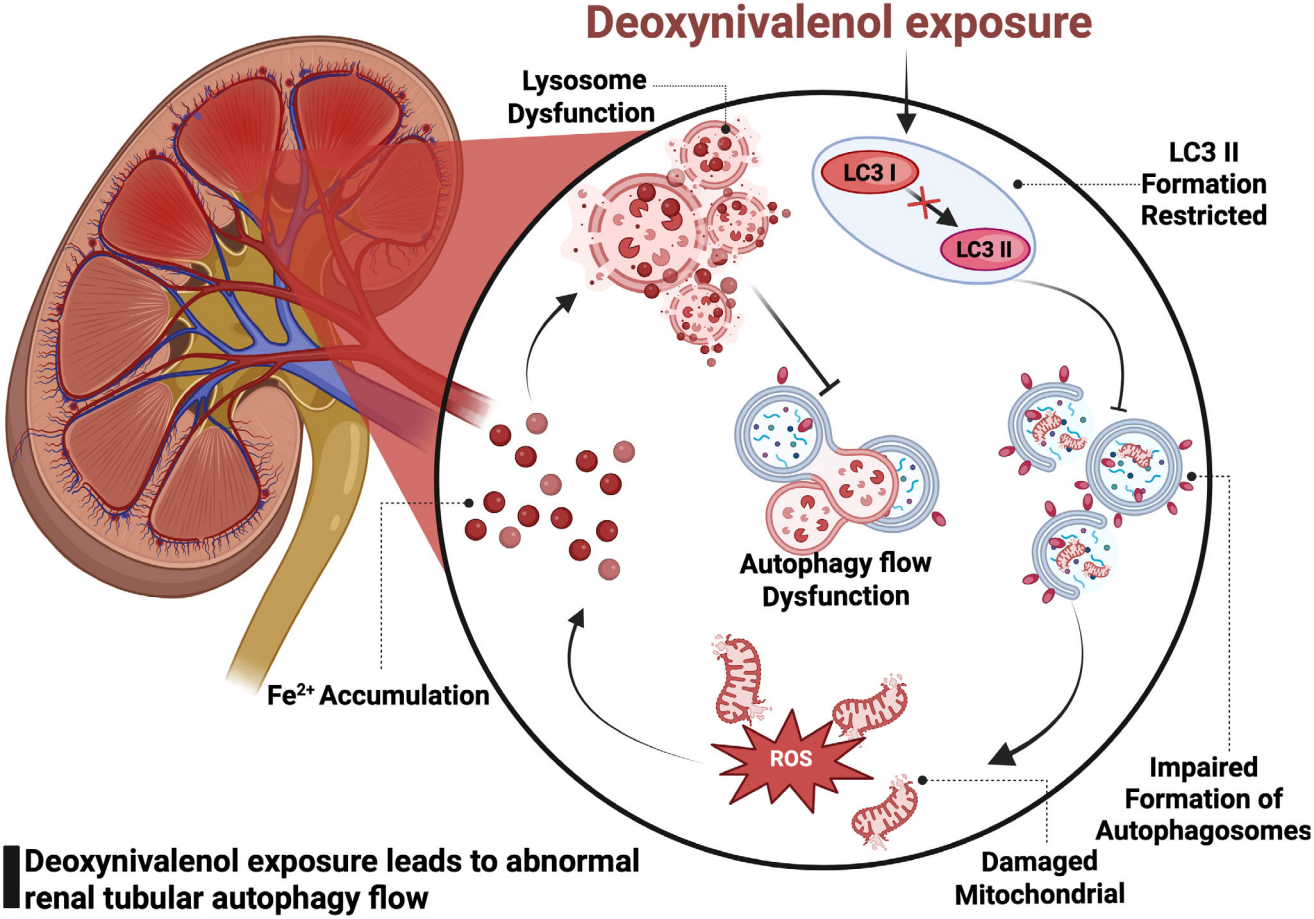
DON exposure leads to impaired autophagosome formation, thereby impairing mitochondrial degradation. Damaged mitochondria increase oxidative stress, reduce ferroptosis resistance, and cause lysosomal Fe^2^⁺ accumulation, further disrupting autophagy. These findings reveal DON's nephrotoxic mechanism and suggest potential therapeutic targets.

In summary, we identified a potential mechanism of renal injury by DON exposure, which could cause renal tubular ferroptosis in vivo and in vitro. We also demonstrated that DON exposure leads to impaired autophagosome formation and interferes with the degradation of damaged mitochondria. This promoted intracellular superoxide production, which reduced the sensitivity of TCMK‐1 to ferroptosis. Excess intracellular superoxide and Fe^2+^ accumulation simultaneously induce lysosomal dysfunction (**Figure** **8**). According to our findings, DON exposure induced multistep impairment of the autophagy flux in TCMK‐1, leading to a vicious cycle. This provided a new insight into the development of effective therapies for renal injury induced by DON exposure.

## 5 Experimental Section

### Chemicals and Reagents

DON (C_15_H_20_O_6_, MW: 296.31) came from NCS Testing Technology Co., LTD. (Beijing, China), which had a purity of ≥ 98%. MitoTempo was from MedChemExpress LLC (USA). For other reagents, refer to the Supporting Information.

### Mouse Models

Changsheng Biological Technology Co., Ltd. (Liaoning, China) provided the male C57BL/6 mice used in this study, which ranged in weight from 14 to 16 g and were 3–4 weeks old. The Animal Experiment Management Regulations (Ministry of Science and Technology of China, 2004) were authorized by the Animal Care and Use Committee of Northeast Agricultural University, China, to regulate the treatment of the study's animals. The experimental ethics authorization number for the C57BL/6 mice was NEAUEC2023 03 73. The mice were housed in a room with a 12‐h light/dark cycle, free access to food and water, a relative humidity of 45%, and an ambient temperature of 25 ± 2 °C.

The C57BL/6 mice were permitted to adjust to their living environment for one week prior to the commencement of all appropriate experiments. To investigate how DON exposure contributes to the cause of kidney injury in mice, the mice were randomly divided into 3 groups: CON, L‐DON, and H‐DON. The maximum DON content in the feed, as determined by the analysis, was 3817.5 µg kg^−1^, while the median was 762.3 µg kg^−1^. The daily DON exposure dose for mice was obtained by converting the maximum and median values of DON content in the feed, the amount of food eaten by the mice per day, and their body weights. The calculated low and high doses for mice exposed to DON were 182.9 and 1923.7 µg kg^−1^ b.w. per day, respectively. To facilitate comparisons with other study,^[^ ^54^ ^]^ the doses were adjusted to 150 and 2300 µg kg^−1^ b.w. Every day for 4 weeks, mice received DON treatment (water was used to dissolve DON). The mice were anesthetized with carbon dioxide and executed by decapitation. Collection of mice serum and kidney tissue for follow‐up experiments.

### Serum Biochemical Index Analysis

Detection of Scr and Urea in mouse serum using the commercial kit (Jiancheng Bioengineering, Nanjing, China). The entire process strictly follows the kit assay instructions provided by the manufacturer. Measurements were made using a multifunctional enzyme labeler (BioTek, USA).

### H&E, Masson, and IHC Staining

The kidneys of mice in each group were fixed in 4% paraformaldehyde for 72 h. The tissue was replaced with fresh paraformaldehyde and deeply fixed for 1 week. Kidneys were dehydrated using gradient ethanol and purified in xylene. Tissue sections (4–5 µm) were made after embedding the tissue in paraffin. Tissue sections were deparaffinized and subjected to H&E staining and Masson staining. After deparaffinization, the sections were successively subjected to antigen repair, incubation with primary antibodies and secondary antibodies conjugated with peroxidase, and finally, DAB was added for color development. The following primary antibodies were utilized: LAMP2 (ABclonal, A1961; 1:100), CTSD (ABclonal, A19680; 1:50). The images were observed and saved using a microscope (DM18, Leica, Germany).

### TEM Analysis

TCMK‐1 was digested using trypsin, and the cells were centrifuged (1200 rpm, 5 min). TCMK‐1 and kidney tissues were fixed using 4% glutaraldehyde. Subsequently, the tissues and cells were fixed using OsO_4_ and embedded using resin. Stain the sections using uranyl acetate and lead citrate. Photographs were taken using a transmission electron microscope (HT7650, Hitachi, Japan).

### MDA, SOD, and CAT Measurement

Kidney tissues were homogenized and prepared for testing. Test for malondialdehyde (MDA) (S0131S, Beyotime, China), superoxide dismutase (SOD) (S0101S, Beyotime, China), and catalase (CAT) (S0051, Beyotime, China) according to the instructions provided by the manufacturer.

### Mass Spectrometry and Bioinformatics

TCMK‐1 is a mouse renal tubular epithelial cell. TCMK‐1 cells from different groups were lysed using SDS lysis buffer. Protein concentrations in each of the groups were determined using the Nano method (Thermo, USA). 100 µg of protein solution was mixed well with pre‐cooled acetone and left at −20 °C for 2 h, and the precipitates were collected by centrifugation (15 000 g, 10 min, 4 °C). After washing the precipitates using methanol, the precipitates were resuspended by adding 8 µL of 8 m urea solution, then diluted to 50 µL with 50 mm NH_4_HCO_3_. Sequencing grade trypsin was added and digested at 37 °C for 18 h. After elution of the peptides, the eluates were concentrated to dryness, and the samples were resuspended by adding 15 µL of 0.1% formic acid. Samples were analyzed by mass spectrometry using timsTOF Pro2 (Bruker, USA). Mass spectrometry measurements were performed using the in‐built “DDA PASEF‐standard_1.1sec_cycletime” method developed by Bruker Daltonics. Data were analyzed using MaxQuant with the Andromeda search engine, and protein identifiers were annotated and assigned using the Uniprot database.

### Cell Culture

Mouse renal tubular epithelial cells (TCMK‐1) were supplied by Servicebio (Wuhan, China). The medium was DMEM with 10% fetal bovine serum and 1% antibiotics (penicillin/streptomycin). Cells were cultured at 37 °C with 5% CO_2_.

### DCFH‐DA Staining

The effect of DON exposure on TCMK‐1 ROS levels was examined using the DCFH‐DA probe (S0033S, Beyotime, China). TCMK‐1 was inoculated on 35 mm dishes and subsequently treated with DON for 48 h. TCMK‐1 were incubated with serum‐free medium containing 10 µm DCFH‐Da for 30 min at 37 °C. Fluorescence intensity was detected using laser confocal microscopy at 488/525 nm (Olympus, Tokyo, Japan).

### Lipid Peroxidation Measurement

The level of TCMK‐1 lipid peroxidation was detected using the Bodipy C11 probe (S0043S, Beyotime, China). TCMK‐1 was inoculated on 35 mm dishes and subsequently treated with DON for 48 h. TCMK‐1 were incubated with 2 µm Bodipy C11 for 30 min at 37 °C. Fluorescence intensity was detected using laser confocal microscopy at 581/591 nm or 488/510 nm (Olympus, Tokyo, Japan).

### AFM Morphological Analysis

In all AFM experiments, cells were spread on poly‐l‐lysine‐coated Confocal dishes. The coated poly‐l‐lysine was for better cell attachment to the glass slides, thus enabling high‐resolution AFM imaging. Different groups of TCMK‐1 were treated with DON, washed three times with PBS, and subsequently fixed by adding 4% paraformaldehyde for 10 min. Topographic images were acquired using the NanoWizard 4 (Bruker, USA) in JPK QI mode using the ATEC‐CONT cantilevers. The tip height of these pointed cantilevers (radius of curvature less than 10 nm) was increased (15 µm) to avoid cantilever contact with the cell during imaging. Results were analyzed using Gwyddion (Version 2.67).

### Cellular Labile Iron Detection

Fe^2+^ concentration within TCMK‐1 was measured using the FerroOrange probe (F374, Dojindo, Japan). TCMK‐1 was inoculated on 35 mm dishes and subsequently treated with DON for 48 h. TCMK‐1 were incubated with 1 µm FerroOrange for 30 min at 37 °C. Fluorescence intensity was detected using laser confocal microscopy at 543/570–620 nm (Olympus, Tokyo, Japan).

### Cytoskeleton Immunofluorescence Imaging

The morphology of microfilaments and microtubules in TCMK‐1 was detected using the Actin probe (C2201S, Beyotime, China) and Tubulin probe (C1050, Beyotime, China), respectively. TCMK‐1 was inoculated on 35 mm dishes and subsequently treated with DON for 48 h. Cells were fixed using 4% paraformaldehyde for 20 min, immediately followed by washing the cells four times using PBS containing 1% Triton. Actin and Tubulin staining were performed separately for TCMK‐1 according to the instructions provided by the manufacturer. Fluorescence intensity was detected using laser confocal microscopy at 488/518 nm or 555/565 nm (Olympus, Tokyo, Japan).

### Autophagy Flux Analysis

Cellular autophagy status was detected by infecting TCMK‐1 with an adenovirus (C3012, Beyotime, China) expressing the mCherry‐GFP‐LC3B fusion protein. TCMK‐1 were infected at 10 MOI for 24 h. TCMK‐1 was inoculated on 35 mm dishes and subsequently treated with DON for 48 h. Fluorescence intensity was detected using laser confocal microscopy at 488/518 nm or 587/610 nm (Olympus, Tokyo, Japan).

### Detection of MitoSOX Generation and Mitochondria Staining

Mitochondrial superoxide changes in TCMK‐1 were detected using a MitoSOX probe (S0061S, Beyotime, China). Mitochondria were also localized using the MitoTracker probe (C1048, Beyotime, China). TCMK‐1 was inoculated on 35 mm dishes and subsequently treated with DON for 48 h. TCMK‐1 were incubated with 5 µm MitoSOX for 30 min at 37 °C. After changing the medium, the TCMK‐1 were incubated with 200 nm MitoTracker for 30 min at 37 °C. Fluorescence intensity was detected using laser confocal microscopy at 510/580 nm or 490/516 (Olympus, Tokyo, Japan).

### Detection of Mitochondrial Membrane Potential

Mitochondrial membrane potential changes in TCMK‐1 were detected using the JC‐1 probe (C2003S, Beyotime, China). TCMK‐1 was inoculated on 35 mm dishes and subsequently treated with DON for 48 h. TCMK‐1 were incubated with JC‐1 dyeing solution for 20 min at 37 °C. Wash 2 times using the staining buffer after removing the dyeing solution. Fluorescence intensity was detected using laser confocal microscopy at 490/530 nm or 525/590 (Olympus, Tokyo, Japan).

### Lysosomes Staining

Lysosomes were localized using the LysoTracker probe (C1047S Beyotime, China). TCMK‐1 was inoculated on 35 mm dishes and subsequently treated with DON for 48 h. TCMK‐1 were incubated with 50 nm LysoTracker for 10 min at 37 °C. Fluorescence intensity was detected using laser confocal microscopy at 504/511 nm (Olympus, Tokyo, Japan).

### Western Blot Assay

Briefly, proteins were electrophoresed on Tris‐glycine acrylamide gels and subsequently transferred to an NC membrane. Antigen blocking was performed for 10 min using 1% PVP40000. The NC membranes were incubated overnight at 4 °C using the primary antibody and then incubated for 1 h at room temperature using the secondary antibody. The following primary antibodies were utilized: GAPDH (Servicebio, GB15004‐100; 1:2000), 4HNE (ABclonal, A24456; 1:5000), KIM‐1 (ABclonal, A2831; 1:1000), SLC3A2 (ABclonal, A24735; 1:2000), FTL (Proteintech, 10727‐1‐AP; 1:5000), ATG4B (ABclonal, A5059; 1:2000), LC3B (Proteintech, 23274‐1‐AP; 1:1000), TOMM20 (Wanlei, WL02512; 1:1000), VDAC3 (ABclonal, A26244; 1:1000), LAMP1 (ABclonal, A21194; 1:10 000), LAMP2 (ABclonal, A1961; 1:3000), CTSB (ABclonal, A19005; 1:1000), CTSD (ABclonal, A19680; 1:4000).

### DON Content Measurement

5 g of collected complete pig feed, corn, wheat bran, and soybean meal samples were crushed and sieved. The samples were added to 50 mL of a methanol and water (1:9) mixture. The mixtures were filtered, and the filtrates were collected. The DON concentrations in the filtrates were measured using a DON ELISA kit (Youlong, BA0941, China). Follow up according to the instructions provided by the manufacturer.

### Statistical Analysis

GraphPad Prism (version 9, San Diego, USA) was used to analyze and illustrate the data. The values were presented as mean ± S.D. Comparisons among the groups were evaluated using a one‐way ANOVA with the Tukey test. A statistically significant difference was defined as a *P* value < 0.05.

### Ethics Statement

Animal care in this study was performed in accordance with the Animal Experiment Management Regulations (Ministry of Science and Technology of China, 2004), approved by the Animal Care and Use Committee of Northeast Agricultural University, China.

## Conflict of Interest

The authors declare no conflict of interest.

## Author Contributions

H.C. conceptualized the project and designed the experiments. H.C. and X.Z. performed the experiments. H.C. performed the analysis of the data. H.C. and J.M. supervised the project and acquired the funding. All the authors read and approved the final manuscript.

## Supporting information



Supporting Information

## Data Availability

The data that support the findings of this study are available from the corresponding author upon reasonable request.

## References

[1] Z. Zhu , W. Guo , H. Cheng , H. Zhao , J. Wang , M. F. Abdallah , X. Zhou , H. Lei , W. Tu , H. Wang , J. Yang , J. Hazard. Mater. 2024, 474, 134695. 38815395 10.1016/j.jhazmat.2024.134695

[2] B. Murtaza , L. Wang , X. Li , M. Y. Nawaz , M. K. Saleemi , A. Khatoon , X. Yongping , Chem. Biol. Interact. 2024, 387, 110799. 37967807 10.1016/j.cbi.2023.110799

[3] H. Chen , W. Xin , J. Jiang , A. Shan , J. Ma , J. Hazard. Mater. 2024, 468, 133854. 38401214 10.1016/j.jhazmat.2024.133854

[4] K. Ma , Y. Bai , J. Li , Z. Ren , J. Li , J. Zhang , A. Shan , Food Funct. 2022, 13, 3905. 35285834 10.1039/d2fo00185c

[5] B. Jia , H. Lin , S. Yu , N. Liu , D. Yu , A. Wu , J. Hazard. Mater. 2023, 451, 131172. 36907058 10.1016/j.jhazmat.2023.131172

[6] F. Chen , G. Yang , H. Qiu , S. Gao , L. Hou , J. Dong , P. Zhao , W. Dong , Poult. Sci. 2025, 104, 105052. 40120248 10.1016/j.psj.2025.105052 PMC11987648

[7] Yu Sun , J. Jiang , P. Mu , R. Lin , J. Wen , Y. Deng , Arch. Toxicol. 2022, 96, 2639. 35900469 10.1007/s00204-022-03337-8

[8] Q. Zhao , S. Zhang , W. Feng , A. Zhou , L. Shi , J. Zhang , Ecotoxicol. Environ. Saf. 2024, 286, 117243. 39447294 10.1016/j.ecoenv.2024.117243

[9] G. Filomeni , D. De Zio , F. Cecconi , Cell Death Differ. 2015, 22, 377. 25257172 10.1038/cdd.2014.150 PMC4326572

[10] D. Glick , S. Barth , K. F. Macleod , J. Pathol. 2010, 221, 3. 20225336 10.1002/path.2697 PMC2990190

[11] M. B. E. Schaaf , T. G. Keulers , M. A. Vooijs , K. M. A. Rouschop , FASEB J. 2016, 30, 3961. 27601442 10.1096/fj.201600698R

[12] Y. Ichimura , T. Kirisako , T. Takao , Y. Satomi , Y. Shimonishi , N. Ishihara , N. Mizushima , I. Tanida , E. Kominami , M. Ohsumi , T. Noda , Y. Ohsumi , Nature 2000, 408, 488. 11100732 10.1038/35044114

[13] C. Sun , Y. Chen , Q. Gu , Y. Fu , Y. Wang , C. Liu , H. Xie , Y. Liao , Z. Zheng , P. Liu , M. Li , Autophagy 2024, 20, 645. 38146933 10.1080/15548627.2023.2299514 PMC10936621

[14] J. Durgan , A. H. Lystad , K. Sloan , S. R. Carlsson , M. I. Wilson , E. Marcassa , R. Ulferts , J. Webster , A. F. Lopez‐Clavijo , M. J. Wakelam , R. Beale , A. Simonsen , D. Oxley , O. Florey , Mol. Cell 2021, 81, 2031. 33909989 10.1016/j.molcel.2021.03.020 PMC8122138

[15] Y. Zhou , Z. Wang , Y. Huang , C. Bai , X. Zhang , M. Fang , Z. Ju , B. Liu , J Mol Cell Biol 2022, 13, 853. 34562084 10.1093/jmcb/mjab059 PMC8800521

[16] H. Nakatogawa , J. Ishii , E. Asai , Y. Ohsumi , Autophagy 2012, 8, 177. 22240591 10.4161/auto.8.2.18373

[17] R. Scherz‐Shouval , E. Shvets , E. Fass , H. Shorer , L. Gil , Z. Elazar , EMBO J. 2007, 26, 1749. 17347651 10.1038/sj.emboj.7601623 PMC1847657

[18] W. Li , Z. Xiang , Y. Xing , S. Li , S. Shi , Cell Death Dis. 2022, 13, 308. 35387983 10.1038/s41419-022-04770-4 PMC8986825

[19] L. W. Thomas , M. Ashcroft , Cell. Mol. Life Sci. 2019, 76, 1759. 30767037 10.1007/s00018-019-03039-y PMC6453877

[20] H. T. Endale , W. Tesfaye , T. A. Mengstie , Front. Cell. Dev. Biol. 2023, 11, 1226044. 37601095 10.3389/fcell.2023.1226044 PMC10434548

[21] T. Kurz , J. W. Eaton , U. T. Brunk , Int. J. Biochem. Cell. Biol. 2011, 43, 1686. 21907822 10.1016/j.biocel.2011.08.016

[22] J. D. Mancias , X. Wang , S. P. Gygi , J. W. Harper , A. C. Kimmelman , Nature 2014, 509, 105. 24695223 10.1038/nature13148 PMC4180099

[23] Z. Zhu , J. Wang , H. Cheng , H. Zhao , C. Liu , X. Zhou , J. Yang , J. Agric. Food Chem. 2025, 73, 2573. 39818813 10.1021/acs.jafc.4c11077

[24] K. Miki , M. Yagi , D. Kang , Y. Kunisaki , K. Yoshimoto , T. Uchiumi , iScience 2024, 27, 109735. 38706843 10.1016/j.isci.2024.109735 PMC11067335

[25] P. Cai , S. Liu , Y. Tu , T. Shan , Sci. Total Environ. 2024, 911, 168648. 37992844 10.1016/j.scitotenv.2023.168648

[26] M. Frangiamone , Á. Lázaro , A. Cimbalo , G. Font , L. Manyes , Food Chem. 2024, 447, 138909. 38489879 10.1016/j.foodchem.2024.138909

[27] M. W. Sumarah , J. Agric. Food Chem. 2022, 70, 9619. 35912482 10.1021/acs.jafc.2c03690

[28] J. D. Miller , Mycotoxins, Climate Change, and Food System Management: A Focus on Cereals, Carleton University, Ottawa, Canada 2022.

[29] A. R. Son , S. Y. Shin , Y. S. Song , B. Hong , B. G. Kim , Anim. Biosci. 2024, 37, 1614. 39164088 10.5713/ab.24.0177 PMC11366530

[30] L. Zhao , L. Zhang , Z. Xu , X. Liu , L. Chen , J. Dai , N. A. Karrow , L. Sun , J. Anim. Sci. Biotechnol. 2021, 12, 74. 34243805 10.1186/s40104-021-00603-0 PMC8272344

[31] T. Goyarts , S. Dänicke , H. Valenta , K.‐H. Ueberschär , Food Addit. Contam. 2007, 24, 369. 17454110 10.1080/02652030600988038

[32] C. J. Amuzie , J. R. Harkema , J. J. Pestka , Toxicology 2008, 248, 39. 18433975 10.1016/j.tox.2008.03.005

[33] D. Wan , L. Huang , Y. Pan , Q. Wu , D. Chen , Y. Tao , Xu Wang , Z. Liu , J. Li , L. Wang , Z. Yuan , J. Agric. Food Chem. 2014, 62, 288. 24341775 10.1021/jf4047946

[34] Y. Tu , S. Liu , P. Cai , T. Shan , Compr. Rev. Food Sci. Food Saf. 2023, 22, 3951. 37421323 10.1111/1541-4337.13203

[35] S. A. Lee , M. Cozzi , E L. Bush , H. Rabb , Am. J. Kidney Dis. 2018, 72, 846. 29866457 10.1053/j.ajkd.2018.03.028 PMC6252108

[36] T. Maruyama , N. N. Noda , J. Antibiot. 2017, 71, 72. 10.1038/ja.2017.104 PMC5799747 28901328

[37] Y. Fu , L. Hong , J. Xu , G. Zhong , Q. Gu , Q. Gu , Y. Guan , X. Zheng , Qi Dai , X. Luo , C. Liu , Z. Huang , X.‐M. Yin , P. Liu , M. Li , Autophagy 2019, 15, 295. 30176161 10.1080/15548627.2018.1517073 PMC6333450

[38] D. Akin , S. K. Wang , P. Habibzadegah‐Tari , B. Law , D. Ostrov , M. Li , X.‐M. Yin , J.‐S. Kim , N. Horenstein , W. A. Dunn , Autophagy 2014, 10, 2021. 25483883 10.4161/auto.32229 PMC4502682

[39] M. Li , Y. Hou , J. Wang , X. Chen , Z.‐M. Shao , X.‐M. Yin , J. Biol. Chem. 2011, 286, 7327. 21177865 10.1074/jbc.M110.199059 PMC3044989

[40] J. Yuan , D. Ofengeim , Nat. Rev. Mol. Cell Biol. 2024, 25, 379. 38110635 10.1038/s41580-023-00689-6

[41] A. Fleming , M. Bourdenx , M. Fujimaki , C. Karabiyik , G J. Krause , A. Lopez , A. Martín‐Segura , C. Puri , A. Scrivo , J. Skidmore , S. M. Son , E. Stamatakou , L. Wrobel , Ye Zhu , A. M. Cuervo , D C. Rubinsztein , Neuron 2022, 110, 935. 35134347 10.1016/j.neuron.2022.01.017 PMC8930707

[42] M. Sato , R. Kusumi , S. Hamashima , S. Kobayashi , S. Sasaki , Y. Komiyama , T. Izumikawa , M. Conrad , S. Bannai , H. Sato , Sci. Rep. 2018, 8, 968. 29343855 10.1038/s41598-018-19213-4 PMC5772355

[43] L. Ma , X. Zhang , K. Yu , X. Xu , T. Chen , Yi Shi , Y. Wang , S. Qiu , S. Guo , J. Cui , Y. Miao , X. Tian , L. Du , Y. Yu , J. Xia , J. Wang , Free Radic. Biol. Med. 2021, 168, 25. 33785413 10.1016/j.freeradbiomed.2021.03.023

[44] P. Xiang , Q. Chen , Le Chen , J. Lei , Z. Yuan , H. Hu , Y. Lu , X. Wang , T. Wang , R. Yu , W. Zhang , J. Zhang , C. Yu , L. Ma , Theranostics 2023, 13, 4993. 37771765 10.7150/thno.87968 PMC10526676

[45] K. Vazdar , S. Skulj , D. Bakaric , D. Margetic , M. Vazdar , Mini Rev. Med. Chem. 2021, 21, 1394. 33402082 10.2174/1389557521666210105110538

[46] R A. Weber , F S. Yen , S. P. V. Nicholson , H. Alwaseem , E C. Bayraktar , M. Alam , R C. Timson , K. La , M. Abu‐Remaileh , H. Molina , K. Birsoy , Mol. Cell 2020, 77, 645. 31983508 10.1016/j.molcel.2020.01.003 PMC7176020

[47] F. Rizzollo , S. More , P. Vangheluwe , P. Agostinis , Trends Biochem. Sci. 2021, 46, 960. 34384657 10.1016/j.tibs.2021.07.003

[48] X. Cao , P. Meng , Y. Shao , G. Yan , J. Yao , X. Zhou , C. Liu , L. Zhang , H. Shu , H. Lu , Front. Mol. Biosci. 2022, 9, 899192. 35573732 10.3389/fmolb.2022.899192 PMC9092021

[49] H. Chen , X. Zhou , J. Ma , A. Shan , J. Hazard. Mater. 2024, 480, 135952. 39341193 10.1016/j.jhazmat.2024.135952

[50] J P. Liuzzi , F. Aydemir , H. Nam , M D. Knutson , R J. Cousins , Proc. Natl. Acad. Sci. U S A 2006, 103, 13612. 16950869 10.1073/pnas.0606424103 PMC1564235

[51] Y. Xing , M.‐M. Wang , F. Zhang , T. Xin , X. Wang , R. Chen , Z. Sui , Y. Dong , D. Xu , X. Qian , Q. Lu , Q. Li , W. Cai , M. Hu , Y. Wang , J.‐L. Cao , D. Cui , J. Qi , W. Wang , Nat. Commun. 2025, 16, 985. 39856099 10.1038/s41467-025-56403-x PMC11760952

[52] Q. Lin , S. Li , H. Jin , H. Cai , X. Zhu , Y. Yang , J. Wu , C. Qi , X. Shao , J. Li , K. Zhang , W. Zhou , M. Zhang , J. Cheng , L. Gu , S. Mou , Z. Ni , Int. J. Biol. Sci. 2023, 19, 1192. 36923942 10.7150/ijbs.80775 PMC10008689

[53] R. Asleh , F. M. Nakhoul , R. Miller‐Lotan , H. Awad , D. Farbstein , N. S. Levy , N. Nakhoul , T. C. Iancu , I. Manov , M. Laue , M. G. Traber , K. M. Lebold , A. P. Levy , Free Radic. Biol. Med. 2012, 53, 779. 22749805 10.1016/j.freeradbiomed.2012.06.015 PMC3600120

[54] J. Jiang , Y. Ruan , X. Liu , J. Ma , H. Chen , J. Agric. Food Chem. 2024, 72, 6660. 38501926 10.1021/acs.jafc.4c00556

