# Supplementary material for: Deoxynivalenol Exposure Leads to Abnormal Renal Tubular Autophagy Flow
Source: Adv Sci (Weinh). 2025 Sep 26;12(47):e05486. doi: 10.1002/advs.202505486 (PMC12713016; doi:10.1002/advs.202505486)
Supplement: Supplementary file 1 — Supporting Information [file ADVS-12-e05486-s001.docx]

**Deoxynivalenol exposure leads to abnormal renal tubular autophagy flow**

**Authors: Hao Chen ^a^, Xintong Zhou ^a^, Jun Ma ^a, b^**

^a^ College of Veterinary Medicine, Northeast Agricultural University, Harbin, 150030, PR China.

^b^ Heilongjiang Provincial Key Laboratory of Pathogenic Mechanism for Animal Disease and Comparative Medicine, Harbin 150030, PR China

**^*^Corresponding Author**

Jun Ma - College of Veterinary Medicine, Northeast Agricultural University, Harbin, 150030, PR China; ORCID: 0000-0002-2686-5673; Email: [majun@neau.edu.cn](mailto:majun@neau.edu.cn)

**Supplemental Materials**

***Chemicals***

Beyotime Biological Technology Co., Ltd. (Shanghai, China) supplied the BCA protein kit (P0010), Cell Counting Kit-8 (C0037), Cell Counting Kit-8 (C0037) and trypsin. Clark Bioscience (USA) supplied fetal bovine serum (FBS). Meilun Biological Technology Co., Ltd. (Dalian, China) provided Dulbecco's Modified Eagle's Medium (DMEM). HE Staining Solution (BL700A) and Modified Masson Trichrome Stain Kit (BL1538A) were from Biosharp (Anhui, China). Liproxstatin-1 and Deferoxamine (DFO) were from MedChemExpress LLC (USA).

***TUNEL Assay***

After dewaxing the paraffin sections, add proteinase K. The paraffin sections and cell smears awaiting examination were further processed according to the operating instructions of the TUNEL detection kit (C1086, Beyotime, China).

***RT-PCR***

TCMK-1 were treated with DON for 48h. Total RNA from cells was extracted using the TRIzol (R0016, Beyotime, China). The RNA samples were reverse transcribed to cDNA using a BeyoRT II kit (D7168L, Beyotime, China). QRTPCR was performed with SYBR Premix Plus (FP205, Tiangen, China). Relative mRNA expressions were determined by 2^-△△Ct^ method. The primers were as follows: LAMP1: Forward CCACCTACGAGACTGCGAAT, Reverse TGGTCACCGTCTTGTTGTCC; LAMP2: Forward CAGTACCTGACAAGGCGACA, Reverse TGAAAGCTGAGCCATTAGCCA; CTSD: Forward CTGCTGGGTCCACCATAAGT, Reverse TTGGCAAAGCCGACCCTATT; CTSB: Forward GGCTCTTGTTGGGCATTTGG, Reverse GGTCTTCAGCAGACACCTCC.

***Determination of ATP and NAD^+^ content***

ATP content and NAD^+^ content in TCMK-1 were detected separately using the kit (S0026/S0175, Beyotime, China). The procedure was performed in strict accordance with the instructions provided by the manufacturer.

**Fig S1**

**
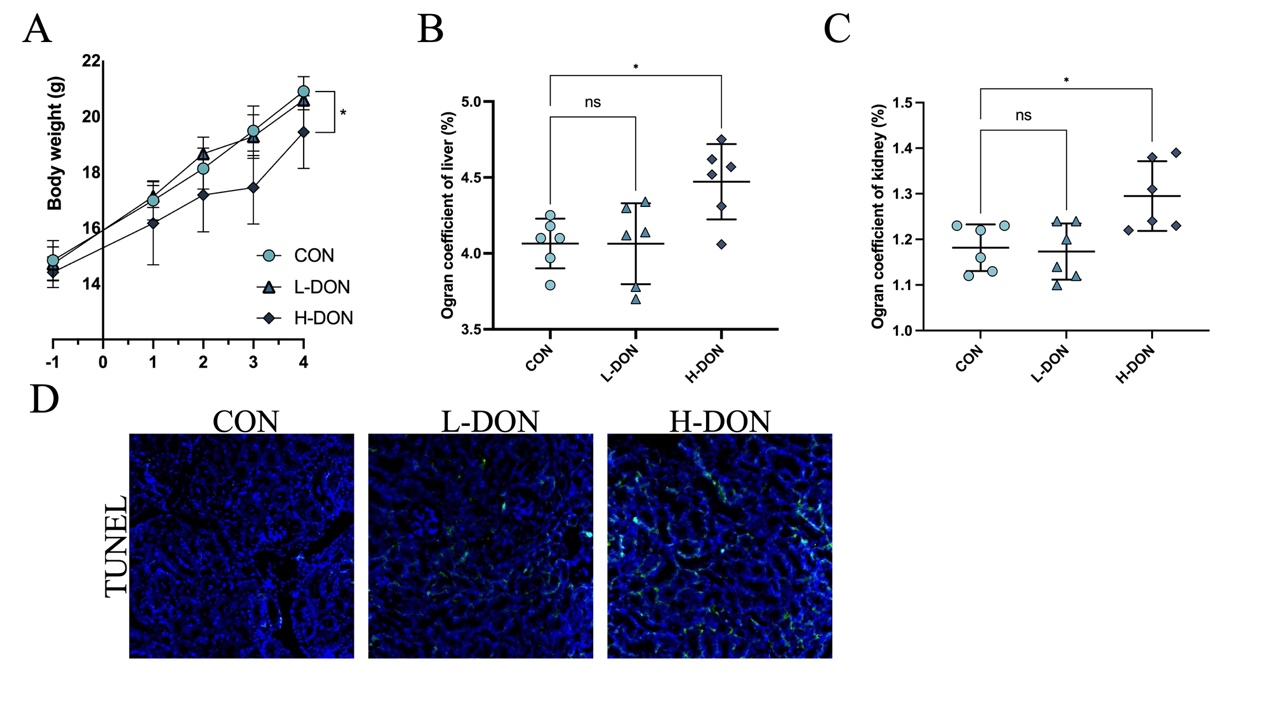
**

**Figure S1** Effects of DON exposure on major organ coefficients in mice (A) Body weight of mice. (B) Ogran coefficient of liver. (C) Ogran coefficient of kidney. (D) Representative TUNEL stained paraffin sections from kidney tissues. The values were expressed as the mean ± S.D. (**P* < 0.05 compared with the control group).

**Fig S2**

**
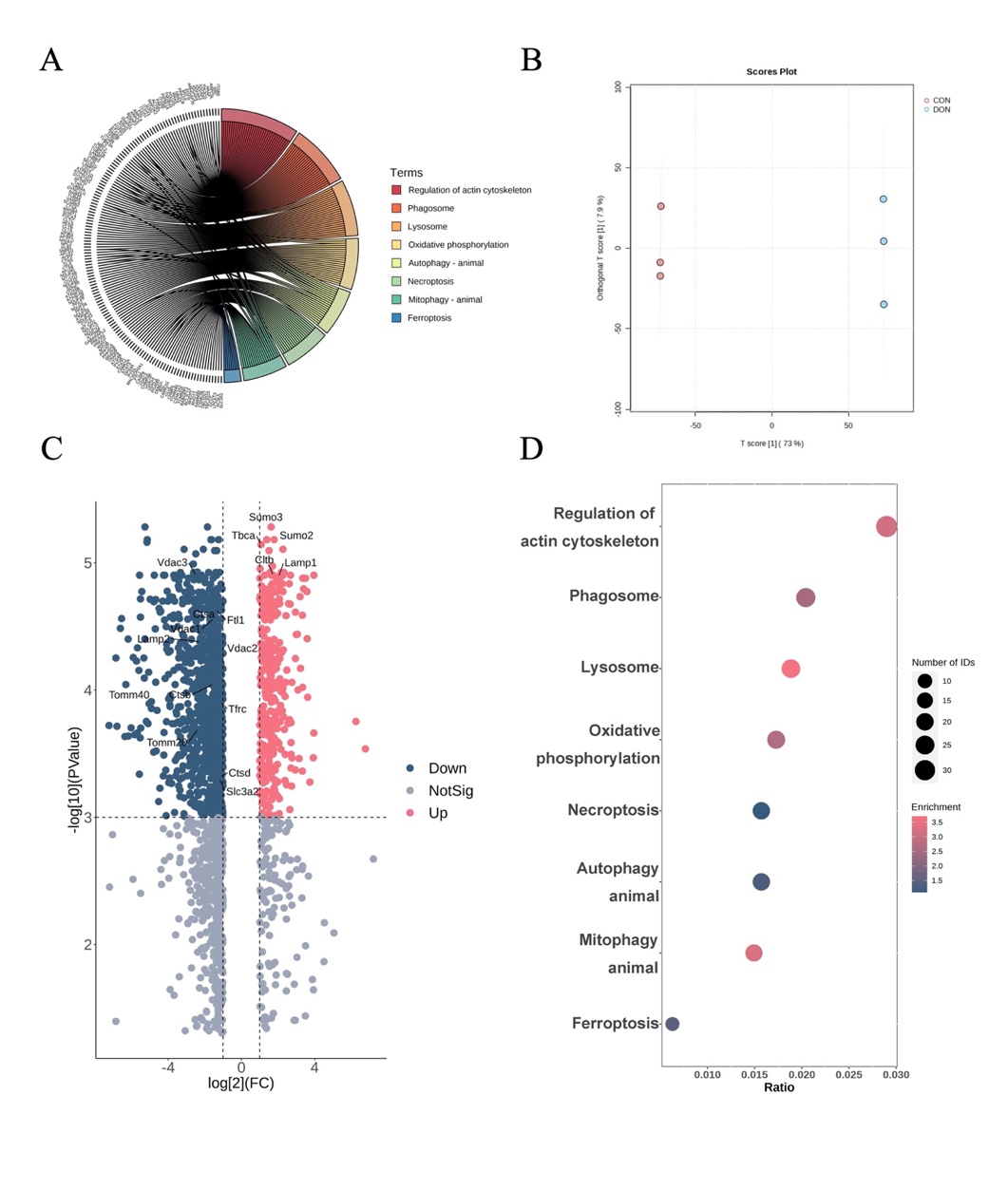
**

**Figure S2** DON treatment of TCMK-1 was followed by proteomic analysis. (A) H-DON and control differential protein classifications. (B) OPLS-DA analysis. (C) Differential protein volcano plot of H-DON and control. (D) KEGG pathway analysis.

**Fig S3**

**
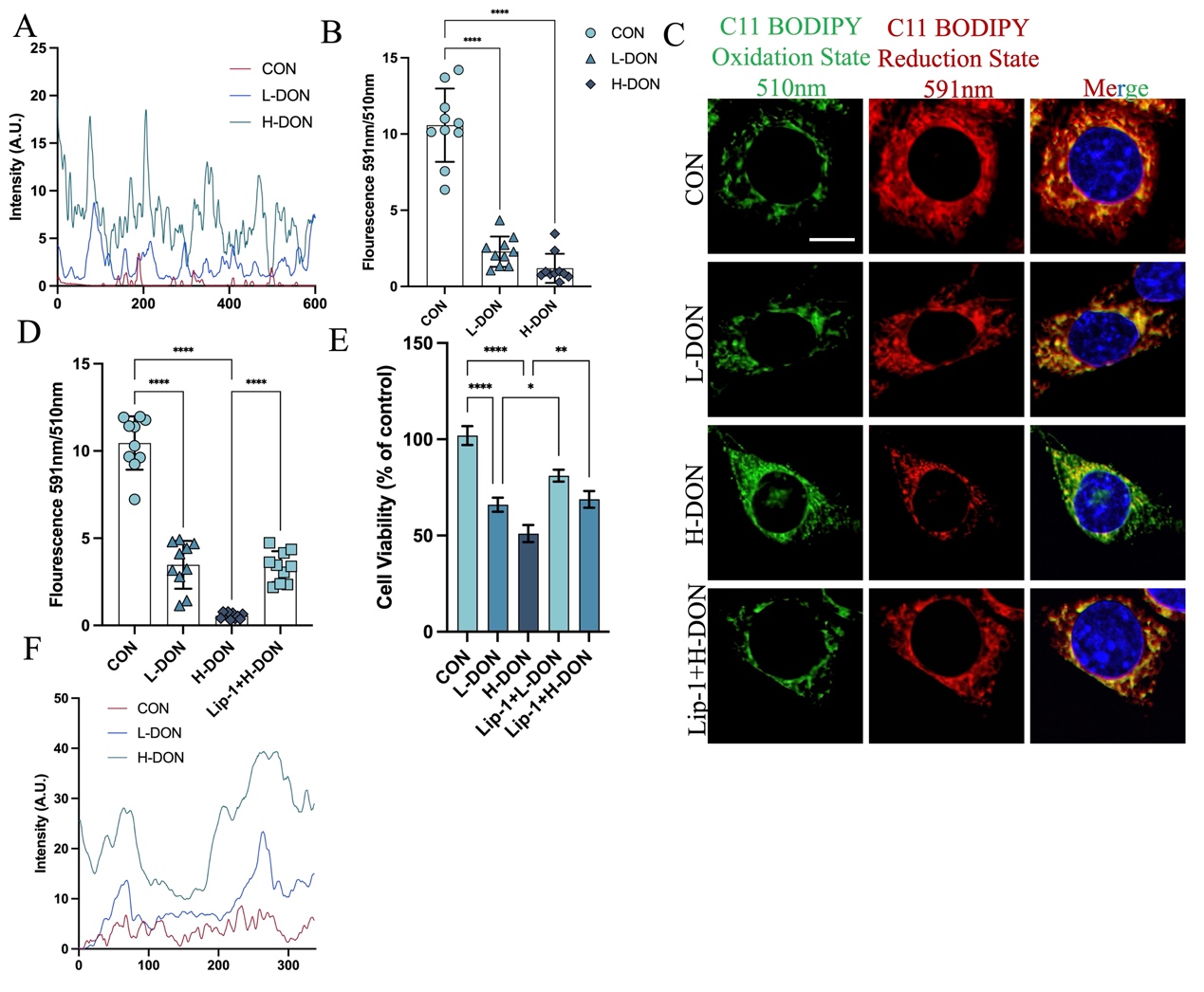
**

**Figure S3** (A) Quantification of DCFH-DA staining in TCMK-1. (B) Quantification of C11 BODIPY staining in TCMK-1. (C) TCMK-1 cells pre-treated with Lip-1 (100 nM) for 2 h, followed by co-treatment with DON for 48 h. Stained TCMK-1 with C11 BODIPY were observed by confocal microscopy (Scale bar = 10 μm). (D) Quantification of C11 BODIPY staining in TCMK-1. (E) TCMK-1 cells pre-treated with Lip-1 (100 nM) for 2 h, followed by co-treatment with DON for 48 h. Cell viability was determined using the CCK-8 assay kit. (F) Quantification of FerroOrange staining in TCMK-1. The values were expressed as the mean ± S.D. (* *P* < 0.05, ** *P* < 0.01 and **** *P* < 0.0001 compared with the control group).

**Fig S4**

**
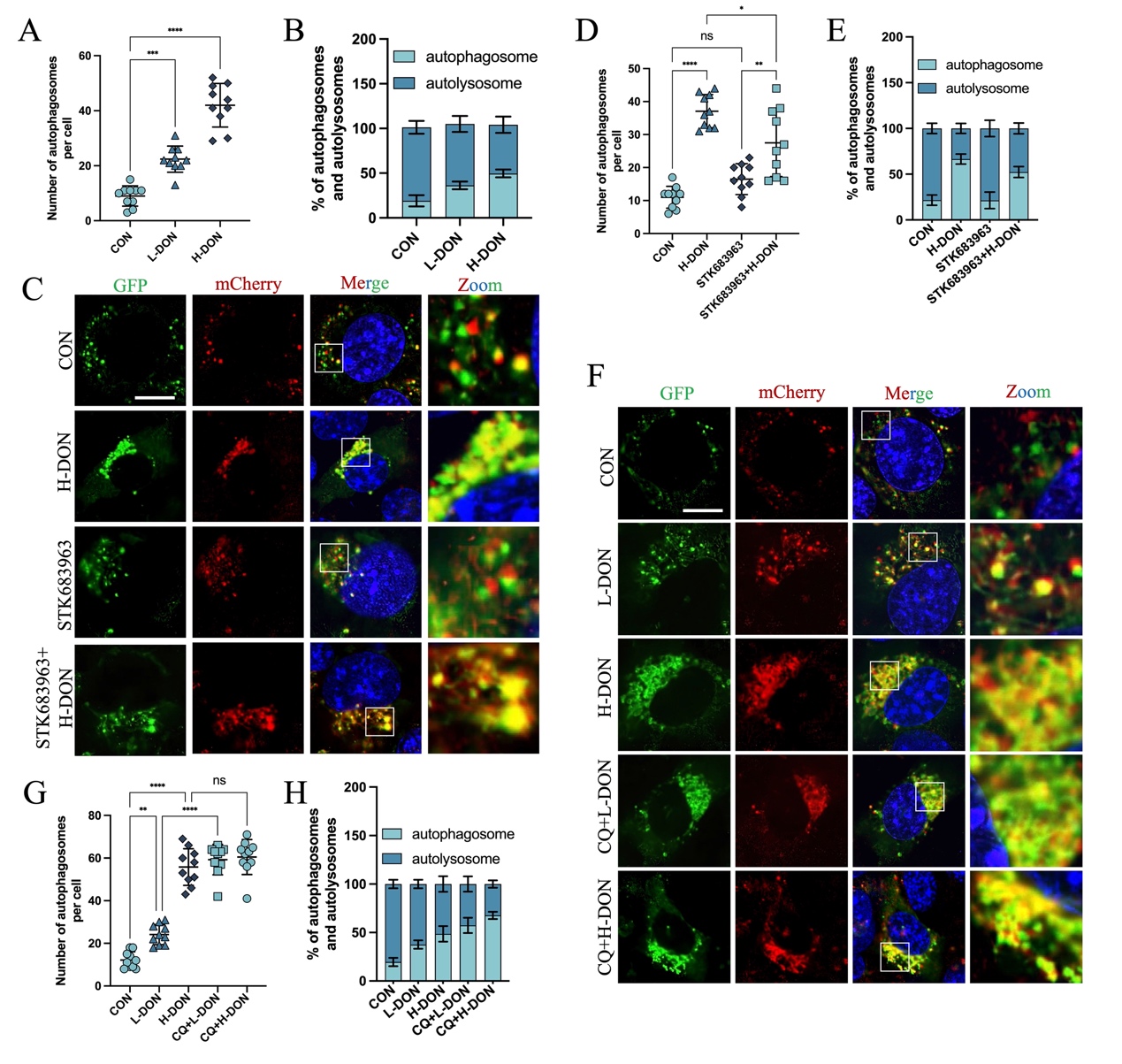
**

**Figure S4** (A-B) Representative images of TCMK-1 transfected Ad-mCherry-GFP-LC3B in the presence of DON treatment. The number of green-red positive puncta in each cell was determined and the ratio of autophagosomes to autolysosomes was calculated. (C-D) TCMK-1 cells pre-treated with STK683963 (25 μM) for 12 h, followed by treatment with DON for 48 h. Representative images of TCMK-1 transfected Ad-mCherry-GFP-LC3B (Scale bar = 10 μm). The number of green-red positive puncta in each cell was determined and the ratio of autophagosomes to autolysosomes was calculated. (F-H) TCMK-1 cells pre-treated with CQ (25 μM) for 2 h, followed by co-treatment with DON for 48 h. Representative images of TCMK-1 transfected Ad-mCherry-GFP-LC3B (Scale bar = 10 μm). The number of green-red positive puncta in each cell was determined and the ratio of autophagosomes to autolysosomes was calculated. The values were expressed as the mean ± S.D. (* *P* < 0.05, ** *P* < 0.1, *** *P* < 0.01 and **** *P* < 0.0001 compared with the control group).

**Fig S5**


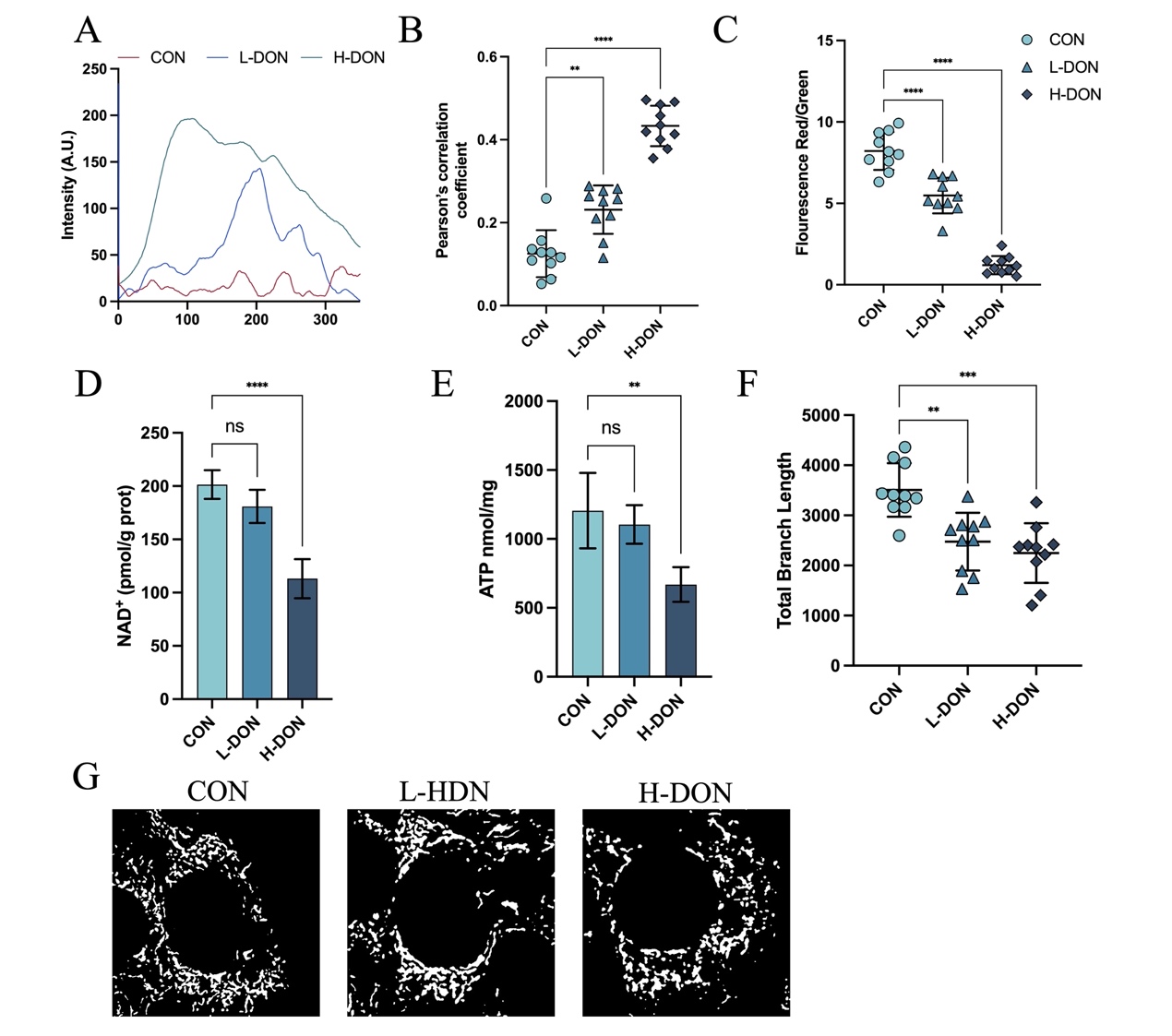


**Figure S5** (A) Quantification of MitoSOX staining in TCMK-1. (B) Quantification of Pearson’s correlation for MitoSOX and MitoTracker. (C) Quantification of JC-1 staining in TCMK-1. (D) The level of NAD^+^ in TCMK-1 were determined by the NAD^+^ assay kit. (E) The level of ATP in TCMK-1 were determined by the ATP assay kit. (F-G) Mitochondrial morphology was analyzed using MiNA (image J plugin). The values were expressed as the mean ± S.D. (** *P* < 0.1, *** *P* < 0.01 and **** *P* < 0.0001 compared with the control group).

**Fig S6**

**
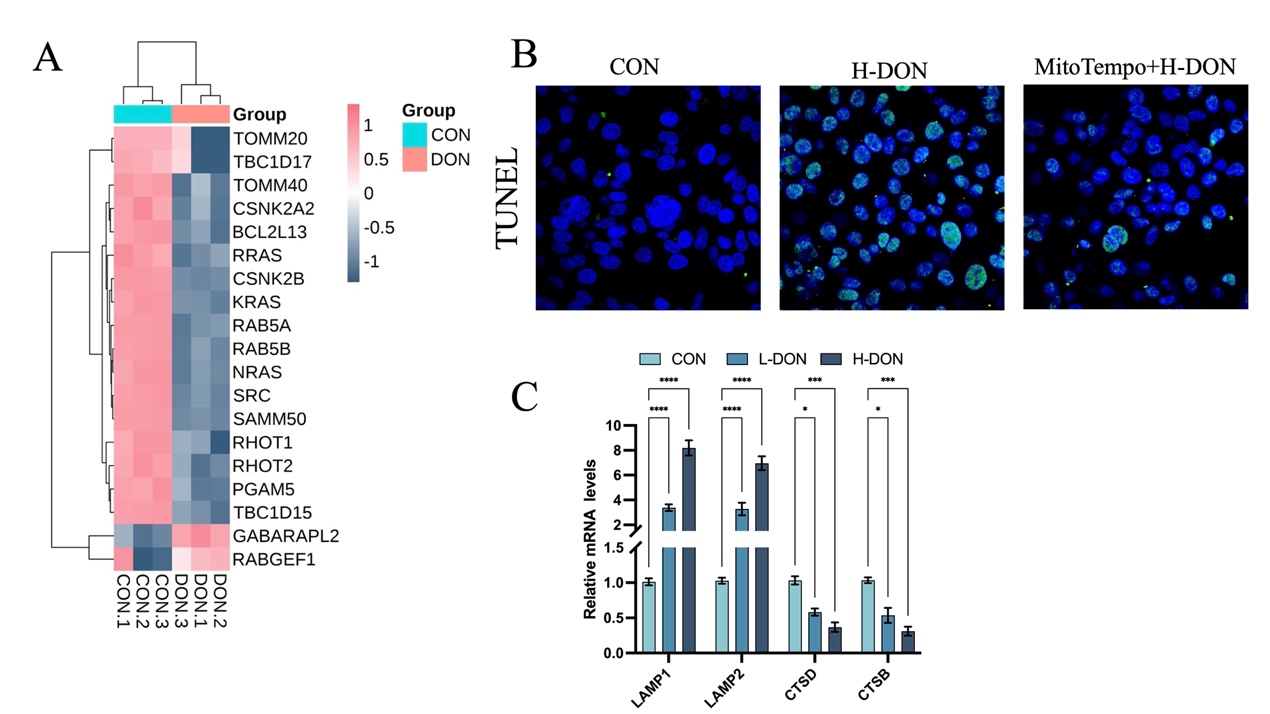
**

**Figure S6** (A) DON treatment of TCMK-1 was followed by proteomic analysis. Heatmap of 19 proteins associated with mitophagy in the H-DON groups compared to the control groups (n=3 technical replicates). (B) TUNEL staining of TCMK-1 cells. (C) Relative mRNA expression of LAMP1, LAMP2, CTSB and CTSD in TCMK-1. The values were expressed as the mean ± S.D. (* *P* < 0.05, *** *P* < 0.01 and **** *P* < 0.0001 compared with the control group).

**Fig S7**

**
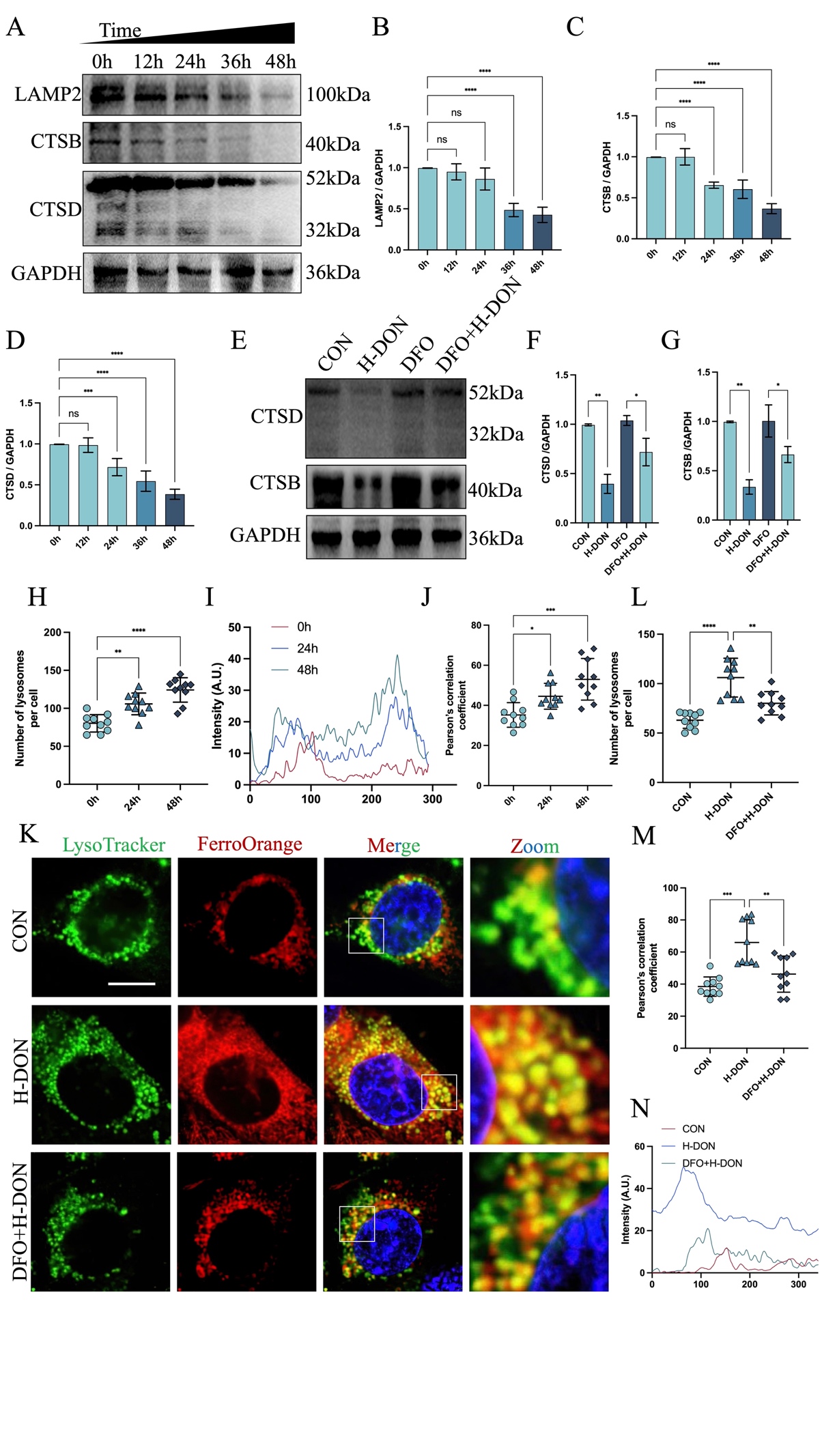
**

**Figure S7** (A-D) TCMK-1 were treated with 200 ng/mL DON for 12h, 24h, 36h or 48h. LAMP2, CTSB and CTSD levels were detected by immunoblotting with antibodies.

(E-G) TCMK-1 were co-treated with DON and DFO (2 μM) for 48h. CTSB and CTSD levels were detected by immunoblotting with antibodies. (H) The number of green positive puncta in each cell were determined. (I) Quantification of FerroOrange staining in TCMK-1. (J) Quantification of Pearson’s correlation for LysoTracker and Fe^2+^. (K-N) TCMK-1 were co-treated with DON and DFO (2 μM) for 48h. Stained TCMK-1 with LysoTracker and FerroOrange were observed by confocal microscopy (Scale bar = 10 μm). The values were expressed as the mean ± S.D. (* *P* < 0.05, ** *P* < 0.01, *** *P* < 0.001 and **** *P* < 0.0001 compared with the control group).
